# Extracorporeal cardiac shock wave stimulation enhances the therapeutic efficacy of intravenously delivered endothelial colony-forming cells via PI3K/AKT signaling in a rat myocardial infarction model

**DOI:** 10.1186/s13287-026-04913-w

**Published:** 2026-02-01

**Authors:** Mingqiang Wang, Dan Yang, Yiming Ma, Yunke Shi, Jinping Lun, Chaoyue Zhang, Xinbin Li, Yuchen Shi, Hongyan Cai

**Affiliations:** 1 Heart Center, The First Affiliated Hospital of Kunming Medical University, Kunming, 650032 Yunnan China; 2 Department of General Internal Medicine, Fuwai Yunnan Hospital, Chinese Academy of Medical Sciences, Affiliated Cardiovascular Hospital of Kunming Medical University, Kunming, 650102 China

**Keywords:** Extracorporeal cardiac shock wave, Myocardial infarction, Endothelial colony-forming cells, Stem cell therapy, PI3K/AKT pathway, Angiogenesis

## Abstract

**Background:**

Extracorporeal cardiac shock wave (ECSW) therapy enhances the function of endothelial colony-forming cells (ECFCs), but whether it can serve as a preconditioning strategy to enhance myocardial infarction (MI) therapy remains unclear. This study investigated the efficacy and mechanism of intravenously delivered ECSW-preconditioned ECFCs (SW-ECFCs) in a rat MI model.

**Methods:**

ECFCs were isolated from the bone marrow of ApoE^-/-^ rats and fully characterized. RNA sequencing of control ECFCs versus SW-ECFCs revealed significant enrichment of the PI3K/AKT pathway. We therefore performed a series of in vitro functional assays on these cells, including Transwell migration, Matrigel tube formation, CCK-8 proliferation, flow cytometric apoptosis analysis, and VEGF-A ELISA. . The role of the PI3K/AKT pathway was interrogated using the inhibitor LY294002. Subsequently, an acute MI model was established in ApoE^-/-^ rats via left anterior descending coronary artery ligation. Rats were randomized into four groups: MI + PBS, MI + ECFCs, MI + SW-ECFCs, and MI + LY294002-pretreated SW-ECFCs (LY-SW-ECFCs), with sham-operated rats as controls. Comprehensive evaluations included echocardiography, serum injury biomarkers, TTC, and histopathological (H&E, Masson) staining, immunohistochemical detection of cardiomyocyte apoptosis and p-eNOS, immunofluorescence assessment of ECFC homing and vascular markers (CD31, α-SMA, VEGF-A), tissue/plasma nitric oxide measurement, and Western blot analysis of PI3K/AKT signaling proteins.

**Results:**

Transcriptomic analysis revealed significant enrichment of the PI3K/AKT pathway in SW-ECFCs. Functionally, ECSW enhanced ECFCs migration, tube formation, proliferation, and VEGF-A secretion, while reducing apoptosis; these effects were largely abolished by PI3K inhibition. In vivo, serum levels of CK, CK-MB, and LDH were significantly elevated in all MI groups compared to the Sham group (*P* < 0.01), indicating comparable initial injury. However, no significant differences were observed among treatment groups (*P* > 0.05). SW-ECFCs transplantation significantly improved cardiac function, reduced infarct size, fibrosis, and apoptosis, and enhanced angiogenesis (*P* < 0.05). These benefits were associated with increased levels of p-AKT, p-eNOS, and BCL-2 protein as well as nitric oxide content, while suppressing the expression of cleaved caspase-3 (*P* < 0.05). Crucially, all these therapeutic benefits were largely abolished by PI3K inhibition.

**Conclusion:**

In conclusion, this study demonstrates that preconditioning ECFCs with ECSW significantly enhances their therapeutic efficacy for myocardial infarction, improving both cardiac function and structural repair. These benefits are mediated primarily through activation of the PI3K/AKT signaling pathway, which augments cell homing, paracrine activity, and survival, thereby providing a novel and promising strategy for cardiac regeneration.

**Supplementary Information:**

The online version contains supplementary material available at 10.1186/s13287-026-04913-w.

## Introduction

 Acute myocardial infarction (AMI), characterized by acute persistent ischemia of major coronary arteries, leading to cardiomyocyte necrosis, remains the leading global cause of mortality [1]. Hypoxia-induced endothelial cell injury represents a fundamental pathogenic mechanism in coronary artery disease progression. Contemporary revascularization strategies, such as percutaneous coronary intervention (PCI) and coronary artery bypass grafting (CABG), significantly improve symptoms and prognosis for many patients by enhancing myocardial perfusion. However, these methods are often insufficient to prevent recurrent myocardial ischemia and significantly improve the quality of life in patients with refractory angina [2, 3]. This unmet clinical need highlights the necessity for innovative treatments, such as stem cell therapies, to stimulate angiogenesis and thereby improve coronary blood flow and prevent further tissue damage.

Stem cell-based regenerative medicine has shown considerable potential for cardiac repair, as evidenced by clinical trials and research that demonstrate its effectiveness in treating heart diseases. Endothelial colony-forming cells (ECFCs), a well-defined population of endothelial progenitor cells derived from bone marrow or peripheral blood, exhibit therapeutic promise owing to their capacity to migrate to ischemic myocardium. At the injury site, ECFCs contribute to vascular repair through direct incorporation into nascent endothelium and/or paracrine-mediated angiogenesis [4–6], highlighting their essential role in post-infarction microvascular reconstitution. However, patients with coronary artery disease exhibit not only a reduced number of ECFCs and impaired migratory capacity [7] but also limited intrinsic myocardial repair ability [8, 9]. Consequently, insufficient recruitment of endogenous ECFCs to the infarcted area hinders endothelial repair, limiting their therapeutic utility. This inherent impairment in vascular repair underscores the necessity of employing exogenous strategies to promote vascular regeneration in ischemic cardiomyopathy. Although targeted delivery of ECFCs (e.g., via infarction border zone transplantation) has shown promising results in treating myocardial infarction, its application is constrained by several challenges [10–12]. Despite encouraging preclinical outcomes, the low engraftment efficiency and poor survival of transplanted ECFCs remain major obstacles to clinical translation [12, 13]. Current preconditioning methodologies, such as hypoxia exposure, genetic modification, and hydrogel encapsulation, often have limited efficacy and involve procedurally complex protocols [14]. Therefore, to advance therapy, it is necessary to develop clinically applicable and safe preconditioning strategies that can robustly enhance the viability, functionality, and regenerative potential of ECFCs.

Extracorporeal cardiac shock wave (ECSW) therapy is a safe, noninvasive treatment modality widely used in cardiovascular diseases [15, 16], with benefits largely ascribed to its pro-angiogenic and perfusion-enhancing effects. At the cellular level, ECSW acts principally through mechanotransduction, a process that transforms mechanical stimuli into biochemical signals. The low-energy pulsed waves produced by ECSW exert shear stress and mechanical forces on cell membranes and the cytoskeleton, thereby activating mechanosensitive ion channels and receptors such as integrins. This initiates rapid intracellular signaling cascades that involve a complex signaling network. The PI3K/AKT pathway is hypothesized to play a pivotal role as an early responder, given its well-documented role in mechanotransduction and cell survival. As an important signaling axis, the PI3K/AKT pathway converts mechanical stimuli into cellular responses that promote survival, proliferation, and angiogenesis. Downstream, phosphorylated AKT activates effector molecules such as eNOS, increasing nitric oxide production to support vasodilation and angiogenesis. This early activation of PI3K/AKT is critical for enhancing cell viability, metabolic activity, and regenerative capacity. Subsequently, the mechanotransductive signal broadens to include upregulation of vascular endothelial growth factor (VEGF), suppression of inflammatory responses, and mobilization of progenitor cells—all collectively contributing to tissue regeneration [17–19]. Notably, ECSW exerts beneficial effects on stem and progenitor cells by positively modulating their survival and function. Clinical evidence indicates that combining ECSW with intracoronary infusion of autologous bone marrow-derived mononuclear cells (BMCs) improves cardiac function in heart failure patients [20]. Previous studies, including those from our group, have identified ECFCs as key cellular targets of ECSW, showing that ECSW enhances ECFC function in vitro, partly through activation of the PI3K/AKT/eNOS pathway [18]. However, the precise therapeutic mechanisms through which ECFCs, whose function is augmented by ECSW-augmented ECFCs ameliorate myocardial infarction remain incompletely defined.

Notably, previous investigations have not directly applied ECSW to stem cells before transplantation, instead focusing on combined therapy. Moreover, conventional cell delivery approaches (intracoronary or intramyocardial injection) have limitations, including technical complexity and restricted feasibility for repeated dosing [21], constraints that could potentially be overcome by intravenous infusion [22]. To date, no study has explored intravenous delivery of ECSW-preconditioned ECFCs. Building on this foundation, we hypothesized that preconditioning with ECSW in vitro could enhance the therapeutic potential of ECFCs by activating the PI3K/AKT pathway, and that these preconditioned cells have shown potential to enhance their homing capabilities and efficacy in cardiac function restoration post-myocardial infarction, as they can modulate the myocardial microenvironment. Therefore, this study was designed to investigate whether ECSW pretreatment can enhance the therapeutic efficacy of intravenously administered ECFCs in a rat myocardial infarction model and to clarify the role of the PI3K/AKT signaling pathway.

## Materials and methods

### Ethics statement and animals

All animal experiments were conducted with the approval of the Institutional Animal Care and Use Committee of Kunming Medical University. All experimental procedures strictly adhered to the ARRIVE guidelines for reporting in vivo experiments [23]. All procedures complied with the Guide for the Care and Use of Laboratory Animals. Coronary heart disease (CHD) is primarily driven by progressive atherosclerosis. ApoE-deficient (ApoE^−/−^) rats spontaneously develop human-like hyperlipidemia and atherosclerosis when maintained on a standard diet [24], making them an ideal model for this study. Therefore, male ApoE^*−/−*^ rats (Suzhou Saiye Biotechnology Co., Ltd.) were utilized in this study. A total of 78 male ApoE^*−/−*^ rats and 3 Sprague-Dawley (SD) rats were used in the study. The rats were housed under controlled conditions: 12-hour light-dark cycle, temperature of 18–22 °C, humidity of 50–70%, with ad libitum access to food and water. At the experimental endpoint, all animals were euthanized by intraperitoneal injection of a lethal dose of 3% pentobarbital sodium.

### Cell culture

Primary bone marrow mononuclear cells (BMCs) were isolated from five 4-week-old ApoE^−/−^ rats and, for comparative purposes, from three age-matched Sprague-Dawley (SD) rats. All rats were anesthetized via intraperitoneal injection of 3% sodium pentobarbital at a dose of 50 mg/kg and then euthanized via cervical dislocation. Long bones were aseptically harvested from each rat, and bone marrow was flushed out. BMCs derived from ApoE^−/−^ and SD rats were processed independently using Ficoll density gradient centrifugation (Solarbio) at 3000 rpm for 30 min at 4 °C. The isolated BMCs were then cultured separately in Endothelial Growth Medium-2 Microvascular (EGM-2 MV; Lonza) supplemented with 10% fetal bovine serum (FBS; Gibco) at 37°C in a humidified 5% CO₂ incubator. After 72 h of culture, non-adherent cells were removed. Subsequently, the medium was refreshed every three days thereafter. Cell morphology and confluence were regularly monitored for both cell types using a phase-contrast microscope(Olympus IX53).

### Identification of ECFCs

After 14 days of culture, ECFCs were characterized using dual fluorescent labeling. Cells were incubated with 20 µg/ml Dil-Ac-LDL (Maokang Biotechnology) for 4 h, fixed in 4% paraformaldehyde for 20 min at room temperature, and then stained with 10 µg/ml FITC-UEA-I for 1 h at 37 °C. Cells double-positive for Dil-Ac-LDL and FITC-UEA-I (Dil-Ac-LDL⁺/FITC-UEA-I⁺) were identified as ECFCs. Immunofluorescence analysis was further performed to confirm ECFC identity. Cells were incubated with primary antibodies against CD133 and CD34, which were detected with APC-conjugated secondary antibodies, and with antibodies against VEGFR2 and CD31, which were detected with FITC-conjugated secondary antibodies. For flow cytometry, cells were incubated with primary antibodies against CD31, CD34, CD133, VEGFR-2, CD14, and CD45 (Bioss Biotechnology), along with APC-labeled goat anti-rabbit IgG, FITC-labeled goat anti-rabbit IgG, and isotype control antibodies. Cells were then washed and resuspended in phosphate-buffered saline (PBS), and the percentages of surface markers on ECFCs were quantified using flow cytometry (BD FACSCelesta).

### ECSW therapy protocol

ECSW therapy was delivered using a MODULITH SLC device (STORZ MEDICAL) according to the following protocol. The device was turned on, initiating a “Warmup” phase on the control panel, followed by an automatic 300-second energy detection cycle. Upon completion, the default parameters were displayed: SCO = 0, energy level = 0.8, frequency = ‘E’ (ECG-triggered), and water cushion height = 1. Before treatment, parameters were adjusted as follows: the energy level was set to 3 (0.09 mJ/mm²), the frequency to 3 Hz, and the coupling pad height to a range of 8.5–9.0 cm. The probe was cautiously advanced to avoid puncturing the coupling pad. The treatment arm was unlocked and then maneuvered to align the probe at a 30° angle relative to the control panel. A coupling accessory was attached to the probe and filled with distilled water to allow the T25 flask to float freely at the water-air interface, ensuring optimal acoustic coupling. Each flask was positioned to maintain the cell monolayer parallel to the probe surface. Therapy was initiated with confirmation of shock wave emission indicated by an audible “click-click” signal. Each session delivered 500 pulses at an energy density of 0.09 mJ/mm² [18, 25, 26].

### RNA sequencing (RNA-seq) analysis

RNA sequencing was performed to identify differentially expressed genes (DEGs) between control ECFCs and SW-ECFCs. The two experimental groups were: (1) Control ECFCs, maintained under standard culture conditions; and (2) SW-ECFCs, treated with extracorporeal cardiac shock waves (ECSW) as described previously. Total RNA was extracted using Trizol reagent. Subsequently, RNA integrity was confirmed by agarose gel electrophoresis, and its quality was assessed on an Agilent 5300 system; only samples with RQN values above the quality threshold were retained. mRNA was enriched with oligo(dT) magnetic beads, then fragmented, and finally reverse-transcribed into cDNA using random primers. Second-strand cDNA was synthesized to generate double-stranded cDNA, which subsequently underwent end repair, 3′ adenylation, and adapter ligation. The constructed libraries were purified, size-selected, PCR-amplified, and sequenced on an Illumina NovaSeq 6000 platform (Shanghai Majorbio Biotechnology Co., Ltd.) in paired-end mode. Bioinformatic analysis was conducted on the Majorbio Cloud Platform (www.majorbio.com). DEGs were identified using DESeq2 with a significance threshold of *p* < 0.05. Functional enrichment analysis of DEGs was performed based on Gene Ontology (GO) and Kyoto Encyclopedia of Genes and Genomes (KEGG) databases to identify significantly associated biological processes and signaling pathways.

### Cellular interventions

ECFCs were randomly assigned to four experimental groups: (1) Control group: maintained under standard culture conditions; (2) ECSW group: treated with extracorporeal cardiac shock waves (ECSW) as described above; (3) LY294002 group: incubated with the PI3K inhibitor LY294002 (100 µM; Selleck) for 6 h; (4) LY294002 + ECSW group: pretreated with 100 µM LY294002 for 6 h before ECSW treatment.

### Cell migration assay

ECFCs from each group were harvested, and 2 × 10⁴ cells suspended in 200 µL medium were seeded into the upper chambers of Transwell inserts (8-µm pore size). The lower chambers contained 400 µL EGM-2 MV medium supplemented with 10% FBS. After 24 h of incubation, the undersides of the Transwell membranes were imaged using an inverted microscope (Olympus IX53, Japan). Migrated cells were quantified using ImageJ software.

### Tube formation assay

Matrigel™ (Corning, 354234, USA) was thawed overnight at 4 °C, then coated onto 96-well plates (50 µL/well) and solidified at 37 °C for 1 h. ECFCs from each group (2 × 10⁴ cells/well in 100 µL medium) were seeded onto the Matrigel™-coated wells. After 4–6 h of incubation, tube formation was observed using an inverted microscope (Olympus IX53, Japan) and quantified using ImageJ software.

### Cell apoptosis assay

ECFCs from each group were harvested, washed with PBS, and resuspended in Annexin V Binding Buffer at a density of 1 × 10⁶ cells/mL (500 µL total volume). Aliquots (100 µL) were incubated with Annexin V-FITC (5 µL; Dojindo Laboratory) and propidium iodide (PI; 5 µL) for 15 min at room temperature in the dark. After adding 400 µL of binding buffer, samples were analyzed by flow cytometry within 1 h.

### Proliferation assays

Cell proliferation was assessed using a Cell Counting Kit-8 (CCK-8; Dojindo Co, Japan) according to the manufacturer’s instructions. ECFCs (2 × 10^3^) from each group were cultured in 96-well plates. Ten microliters of CCK-8 solution was added to each well in the 96-well plates and incubated for 2 h at 37℃ in a 5% CO_2_ atmosphere. The absorbance at 450 nm (OD450) was measured using an enzyme labeling apparatus (Molecular Devices, USA).

### Enzyme-linked immunosorbent assay (ELISA)

The concentration of VEGF-A in cell culture supernatants was quantified using a commercial rat-specific ELISA kit (Shanghai Keaibo Bio, Shanghai, China), according to the manufacturer’s instructions. Before analysis, the supernatants were centrifuged at 1000 × g for 20 min to remove cellular debris and stored at − 80 °C until use. Absorbance was measured at 450 nm using a microplate reader (Thermo Fisher Scientific, MA, USA), and sample concentrations were determined by interpolation from a standard curve.

### Establishment of rat myocardial infarction model

ApoE^-/-^ rats (8-week-old) were anesthetized with 3% sodium pentobarbital (50 mg/kg, intraperitoneally), followed by tracheal intubation and connection to a small-animal mechanical ventilator (90 breaths/min, tidal volume 5 mL, inspiratory: expiratory ratio = 1:1). Under aseptic conditions, a left thoracotomy was performed to expose the heart, and the left anterior descending (LAD) coronary artery was ligated using 6 − 0 polypropylene suture. The thorax was closed in layers, and perioperative antibiotic prophylaxis (e.g., penicillin G, 20,000 U/kg) was administered. Sham-operated rats underwent identical surgical procedures except for LAD ligation. Successful MI induction was confirmed by transient ST-segment elevation on the intraoperative electrocardiogram.

### Animal grouping and treatment strategy

From the cohort that underwent surgery, seven rats were excluded: five due to perioperative mortality and two due to unsuccessful MI induction (assessed by ECG and later TTC staining). Myocardial infarction was further verified by 2,3,5-triphenyltetrazolium chloride (TTC) staining at 24 h post-operation in one randomly selected rat from both the sham-operated and MI-operated groups. After these exclusions, 44 rats with confirmed MI were randomly assigned to four treatment groups (*n* = 11 per group): MI + PBS group: received equal-volume PBS injections at 24 h and 1 week post-surgery; MI + ECFCs group: received intravenous injections of 1 × 10⁶ untreated ECFCs via the tail vein at the same time points; MI + SW-ECFCs group: received intravenous injections of 1 × 10⁶ ECSW-treated ECFCs (SW-ECFCs) at matching intervals; MI + LY294002-SW-ECFCs group: received intravenous injections of 1 × 10⁶ LY294002-pretreated SW-ECFCs at equivalent time points. Sham-operated rats (*n* = 11) served as the surgical control group. No experimental mortality occurred during the study period.

### ECFCs recruitment assay

Twenty-four hours post-MI, an additional nine rats were randomly assigned to an independent group to assess ECFC recruitment, receiving intravenous injections of PKH26-labeled cells from one of three preparations: untreated ECFCs, SW-ECFCs, or LY294002-SW-ECFCs via the tail vein (*n* = 3 per group). Six hours later, rats were anesthetized with 3% sodium pentobarbital and euthanized by cervical dislocation. Cardiac tissues were perfused, fixed, dehydrated, and embedded in OCT medium for cryosectioning. Sections were subjected to immunofluorescence staining with anti-α-actinin antibody (1:200) overnight at 4 °C, followed by incubation with FITC-conjugated secondary antibody (1:300) and DAPI counterstaining. The distribution of labeled ECFCs was analyzed using orthogonal fluorescence microscopy.

### Quantification of serum biomarkers in the acute phase of myocardial injury

Venous blood samples were collected from rats in each group 48 h after myocardial infarction. The whole blood was allowed to clot at room temperature for 2 h and then centrifuged at 3000 rpm and 4 °C for 15 min to separate the serum. The resulting supernatant (serum) was immediately aliquoted for subsequent analysis. Serum levels of creatine kinase (CK), CK-MB, and lactate dehydrogenase (LDH) were measured using commercial assay kits (Changchun Huili) according to the manufacturer’s instructions. The measurements were performed on a fully automated biochemical analyzer employing a dual-reagent method with reagents R1 and R2, after configuring the instrument with the appropriate parameters.

### Echocardiography

Cardiac function was evaluated using a high-frequency echocardiography system (Vevo 2100; VisualSonics, Canada) at 3 weeks postoperatively. Left ventricular end-diastolic diameter (LVIDd), left ventricular end-systolic diameter (LVIDs), and left ventricular ejection fraction (LVEF) were measured from long-axis views and averaged over three consecutive cardiac cycles. Left ventricular end-diastolic volume (LVEDV), left ventricular end-systolic volume (LVESV), and left ventricular fractional shortening (LVFS) were automatically calculated using echocardiographic software. All measurements were performed by an independent investigator blinded to group assignments.

### Triphenyltetrazolium chloride (TTC) staining

Following euthanasia, hearts from each group were rapidly excised and immersed in PBS at 4 °C. The hearts were then briefly chilled at -20 °C for 30 min to facilitate sectioning. Subsequently, they were cut into 2-mm-thick sections and incubated in 1% 2,3,5-triphenyltetrazolium chloride solution at 37 °C for 30 min in a dark water bath, with gentle agitation every 5 min to ensure uniform staining. The sections were then rinsed in PBS for 3–5 min, arranged according to size, and photographed. The infarct area (white) and total area of each section were measured using ImageJ software. The infarct size was expressed as the ratio of the total infarct area across all sections to the total ventricular area of all sections.

### Histologic staining and analysis

Cardiac tissues from all groups were fixed in 4% paraformaldehyde, paraffin-embedded, and sectioned at a thickness of 4 μm. Following deparaffinization and rehydration, sections were stained with hematoxylin and eosin (H&E) and Masson’s trichrome according to the manufacturer’s protocols. Whole-slide images were acquired using a Pannoramic MIDI scanner (3DHISTECH) and analyzed with CaseViewer (3DHISTECH) and ImageJ software.

Apoptosis was assessed using the TUNEL assay (Servicebio), with quantification based on the number of TUNEL-positive cells. For immunohistochemistry, sections underwent antigen retrieval and endogenous peroxidase blockade, followed by blocking with 3% bovine serum albumin (BSA) for 30 min. Sections were incubated with anti-phosphorylated eNOS (p-eNOS) antibody (1:300; Affinity) at 4 °C overnight, rinsed three times with PBS, and then incubated with the secondary antibody (1:200; Affinity) for 50 min at room temperature. Subsequent steps included DAB development, hematoxylin counterstaining, dehydration, and mounting. Images were captured using an Olympus BX53 microscope and analyzed with ImageJ software.

### Immunofluorescence

Apoptosis in cardiac sections was detected using the One-Step TUNEL Apoptosis Detection Kit (Green Fluorescent; Beyotime) according to the manufacturer’s protocol.

For immunofluorescence staining, deparaffinized and rehydrated sections underwent antigen retrieval and endogenous peroxidase blockade, followed by blocking in 3% BSA for 30 min. Slides were incubated overnight at 4 °C with primary antibodies against CD31 (1:200; Abcam, ab222783), α-smooth muscle actin (α-SMA; 1:200; Servicebio, GB111364-100), and VEGF-A (1:200; Affinity, AF5131). After PBS rinses, sections were incubated with corresponding secondary antibodies (1:200; Servicebio; GB22303 for α-SMA and VEGF-A, GB27303 for CD31) for 1 h at room temperature, followed by counterstaining with DAPI for 10 min. Images were acquired using an Olympus BX53 fluorescence microscope and analyzed with ImageJ software.

### Measurement of nitric oxide levels

Plasma and cardiac NO levels were quantified using a Nitric Oxide Assay Kit (Solarbio, BC1475). Cardiac tissues (200 mg) were homogenized in extraction buffer using an ice-bath ultrasonicator, followed by centrifugation at 12,000 rpm for 15 min at 4 °C. Plasma and tissue supernatants (60 µL) were sequentially mixed with Reagent 1 (5 µL), Reagent 2 (10 µL), and Reagent 3 (5 µL), then incubated at 37 °C for 120 min. After adding Reagent 4 (10 µL) and Reagent 5 (10 µL), followed by a 30-minute incubation, 100 µL of chromogenic solution was added, and the mixture was reacted for 10 min at room temperature. Absorbance was measured at 550 nm.

### Western blot analysis

Proteins were extracted from cardiac tissues using RIPA lysis buffer (Beyotime, Cat. No. P0013B) containing 1% phenylmethylsulfonyl fluoride (PMSF; Beyotime, Cat. No. ST507) and 1% protease/phosphatase inhibitor cocktail. Protein concentrations were determined using a bicinchoninic acid (BCA) assay kit (Beyotime, Cat. No. P0012). Proteins were separated by SDS-PAGE and transferred to polyvinylidene difluoride (PVDF) membranes (Millipore, Cat. No. IPVH00010). After blocking in blocking buffer (Beyotime, Cat. No. P0252) for 2 h, membranes were incubated overnight at 4 °C with primary antibodies against Akt (1:1,000; Cell Signaling Technology [CST], #4691), phospho-Akt (1:1,000; CST, #4060), eNOS (1:1,000; CST, #32027), phospho-eNOS (1:250; Millipore, #07-428-I), Bcl-2 (1:1,000; Affinity, AF6139), Cleaved Caspase-3 (1:1,000; CST, #9664), and GAPDH (1:1,000; CST, #5174). Following washes with Tris-buffered saline with Tween-20 (TBS-T), membranes were incubated with horseradish peroxidase (HRP)-conjugated secondary antibody (1:5,000; Affinity, #S0001) for 2 h at room temperature. Protein bands were visualized using enhanced chemiluminescence (ECL) reagent (Millipore, #WBKLS0500) and quantified using ImageJ software, with GAPDH serving as the loading control for normalization.

### Statistical analysis

All data are expressed as the mean ± standard deviation (SD) of at least three independent experiments. For comparisons between two groups, the normality of distribution was assessed using the Shapiro-Wilk test. Data satisfying this assumption were analyzed using the unpaired Student’s t-test, while data violating normality were analyzed using the Mann-Whitney U test. For comparisons among multiple groups, the normality (Shapiro-Wilk test) and homogeneity of variance (Brown-Forsythe test) were verified first. Data satisfying both assumptions were analyzed by one-way analysis of variance (ANOVA) followed by Tukey’s post hoc test. For data that violated the assumption of normality, the Kruskal-Wallis test was applied, followed by Dunn’s post hoc test. For data that passed the normality test but exhibited unequal variances, Welch’s ANOVA was performed, followed by the Games-Howell post hoc test. Investigators were blinded to group assignments during all data acquisition and analysis procedures. Statistical significance was defined as *p <* 0.05. All analyses were performed using GraphPad Prism software (version 9.0).

## Results

### Isolation and characterization of ECFCs from ApoE^-/-^ and SD rats

**Fig. 1:**
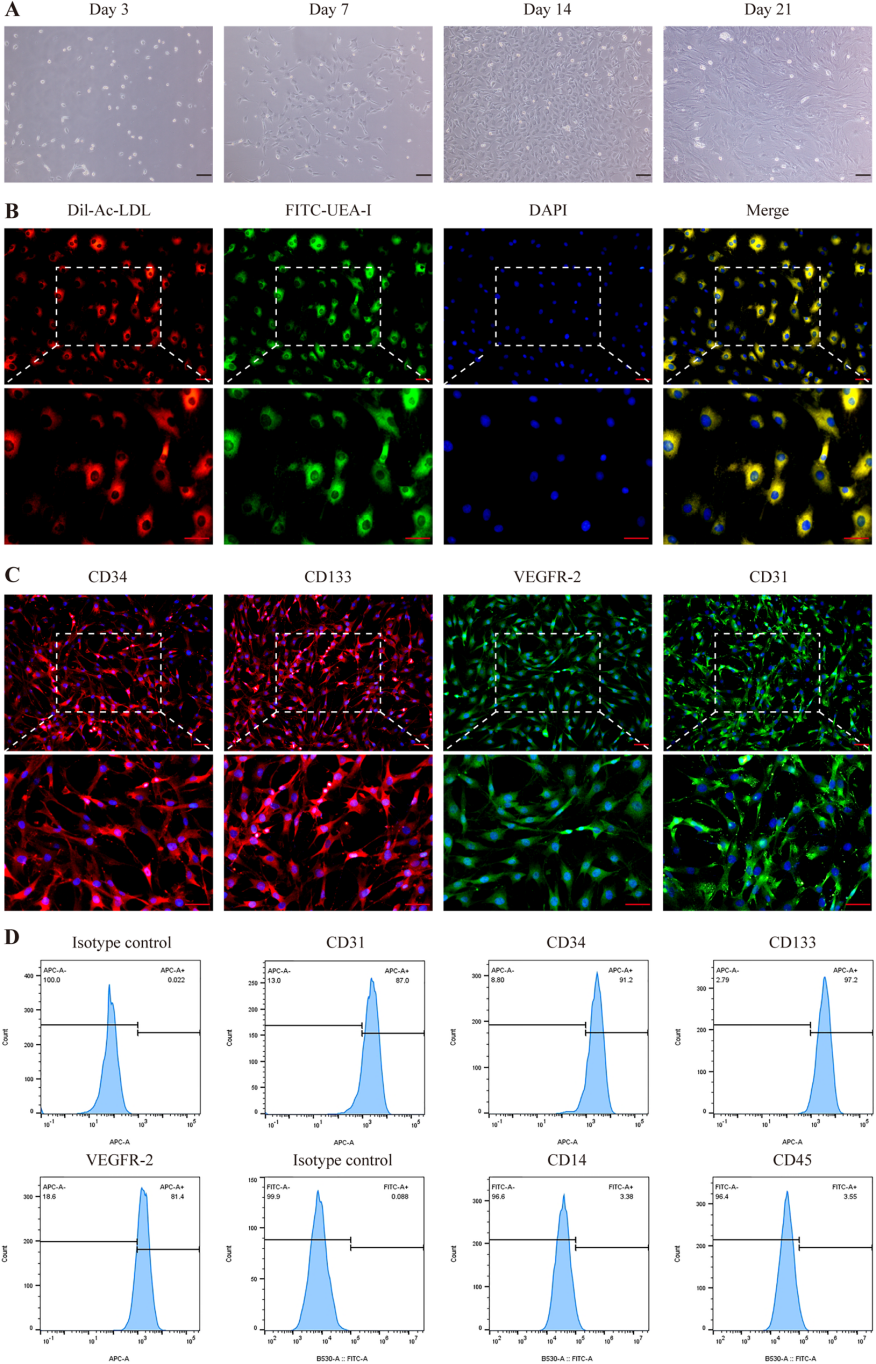
Characterization of rat bone-derived endothelial colony-forming cells (ECFCs). **A** Morphological appearance of ECFCs at days 3, 7, 14, and 21 of culture (scale bar = 100 μm). **B** Fluorescent images showing dual-positive staining for Dil-Ac-LDL (red) and FITC-UEA-1 (green), confirming the endothelial phenotype (scale bar = 50 μm). **C** Immunofluorescence analysis of surface markers (CD31, CD34, CD133, VEGFR-2) (scale bar = 50 μm). **D** Cell surface markers (CD31, CD34, CD133, VEGFR-2, CD14, and CD45) of ECFCs were identified by flow cytometry

ECFCs were successfully isolated and expanded from the bone marrow of ApoE^-/-^ rats. During culture, adherent cells with oval or spindle-shaped morphology emerged by day 3, developed into cobblestone-like monolayers by day 14, and displayed a characteristic endothelial-like morphology by day 21 (Fig. 1A). These cells exhibited dual uptake of Dil-Ac-LDL and binding to FITC-UEA-I (Fig. 1B), and immunofluorescence staining confirmed the positive expression of endothelial markers CD31, CD34, CD133, and VEGFR2 (Fig. 1C). Flow cytometric analysis quantified robust expression of endothelial markers (CD31/VEGFR2: 81–87%; CD34/CD133: 91–98%) with minimal hematopoietic contamination (CD14/CD45: 3–4%) (Fig. 1D). For comparative purposes, ECFCs were also isolated from SD rats. These cells exhibited a highly similar cobblestone morphology, possessed an identical capacity for dual uptake of Dil-Ac-LDL and FITC-UEA-I, and similarly showed high expression of CD31, CD34, CD133, and VEGFR2 (Supplementary Fig. 4A-C). Functional comparison under baseline conditions revealed that wild-type (SD) ECFCs exhibited a trend toward greater migratory and tube-forming capacities compared to ApoE^-/-^ ECFCs, although this difference did not reach statistical significance (Supplementary Fig. 5A-D). This finding prompted us to subsequently focus on enhancing the functional potency of ApoE^-/-^ ECFCs.

###  Transcriptomic analysis identifies activation of the PI3K/AKT pathway by ECSW

**Fig. 2:**
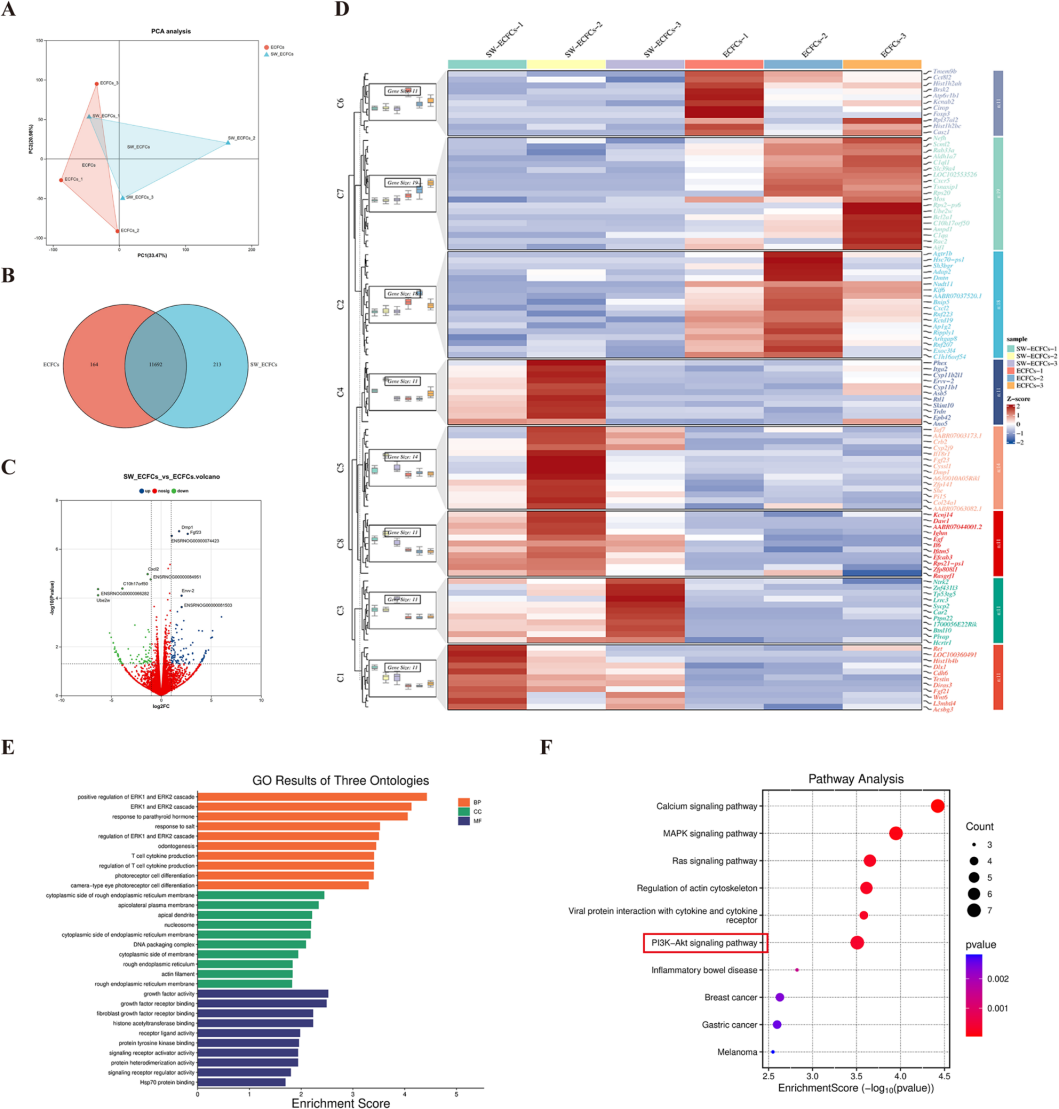
**ECSW preconditioning activates the PI3K/AKT pathway and alters the ECFC transcriptome**. **A** Principal component analysis (PCA) plot demonstrating clear separation along the first principal component following ECSW treatment. **B** Venn diagram depicting the number of genes shared and uniquely expressed in each group. **C** Volcano plot of the 222 differentially expressed genes (DEGs), including 130 upregulated and 92 downregulated genes. **D** Heatmap showing the expression patterns of the identified DEGs. **E** Gene Ontology (GO) enrichment analysis revealed that the identified DEGs were significantly enriched in biological processes related to positive regulation of the ERK1 and ERK2 cascade, molecular functions involving growth factor activity, and cellular components localized to the cytoplasmic side of rough endoplasmic reticulum membrane. **F** Kyoto Encyclopedia of Genes and Genomes (KEGG) pathway enrichment analysis of the DEGs

To investigate the transcriptomic changes induced by ECSW, we performed RNA-seq on control and SW-ECFCs. Principal component analysis (PCA) showed that the first two principal components (PC1 and PC2) accounted for approximately 33.47% and 20.98% of the total variance, respectively. While some overlap was observed, the separation along PC1 indicates that ECSW treatment was a major source of transcriptomic variation (Fig. 2A). Moreover, Venn diagram analysis identified 11,692 genes common to both groups, while 164 and 213 genes were exclusively expressed in the control and SW-ECFCs groups, respectively (Fig. 2B). Differential expression analysis identified 222 significantly dysregulated genes in SW-ECFCs (|log2 fold change| > 1, *p* < 0.05), comprising 130 upregulated and 92 downregulated genes (Fig. 2C). . A volcano plot displays all 222 differentially expressed genes (DEGs). The expression patterns of these DEGs are visualized in a hierarchical clustering heatmap (Fig. 2D), which showed clear separation between the two groups, with upregulated genes predominantly grouped on one side and downregulated genes on the other. KEGG pathway enrichment analysis showed that the DEGs were enriched in several key signaling pathways, including calcium, MAPK, and RAS signaling. Notably, the PI3K-AKT signaling pathway was significantly enriched, particularly among the upregulated DEGs (Fig. 2F). In support of these findings, Gene Ontology (GO) enrichment analysis revealed that the identified DEGs were significantly enriched in biological processes related to positive regulation of ERK1/ ERK2 cascade, molecular functions involving growth factor activity and cellular components localized to the cytoplasmic side of rough endoplasmic reticulum membrane (Fig. 2E).

### ECSW enhances ECFCs’ function and VEGF-A secretion via the PI3K/AKT pathway 

**Fig. 3:**
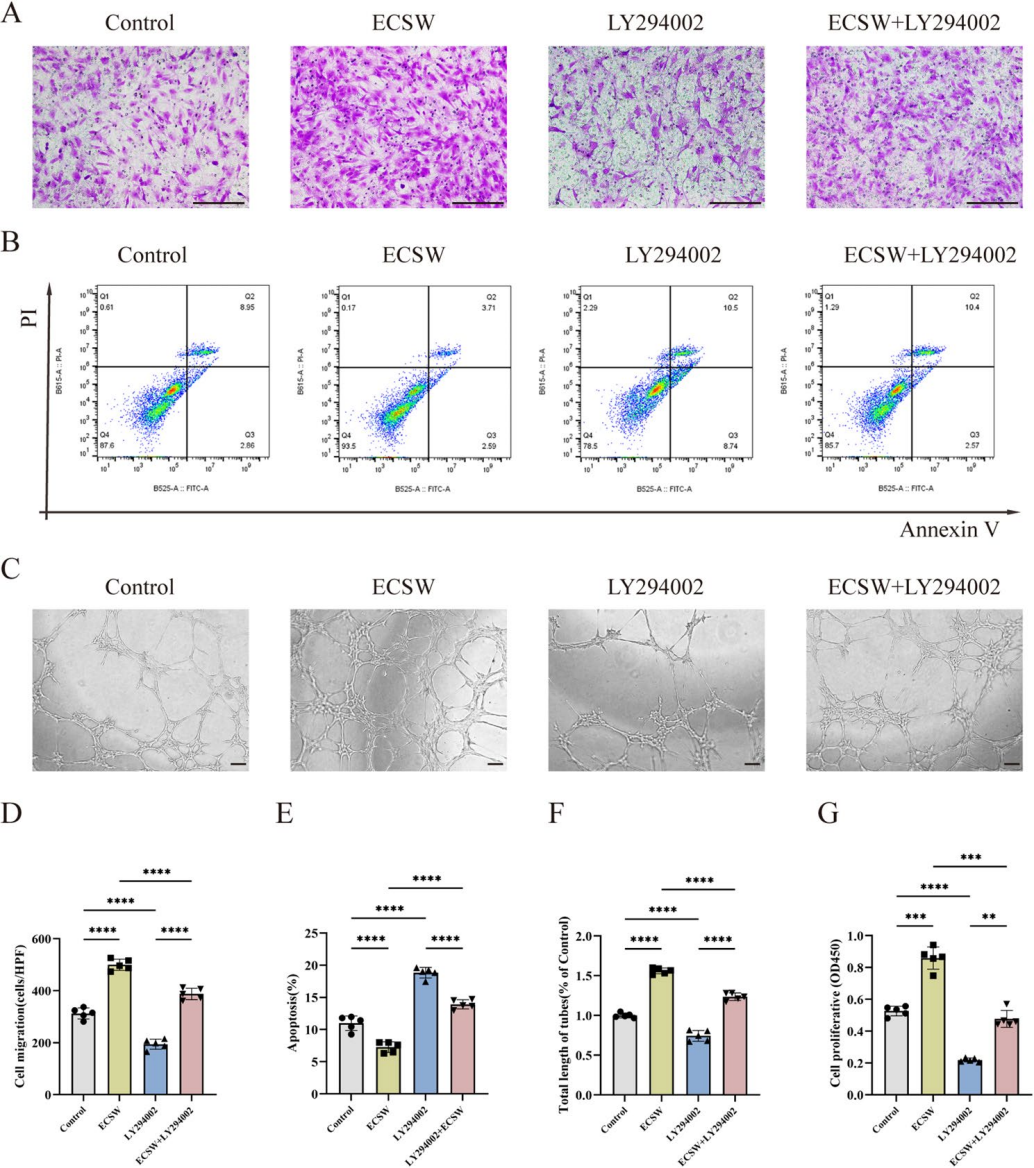
**ECSW enhances ECFC migration, proliferation, tube formation, and apoptosis resistance via the PI3K/AKT pathway**. **A**–**G** Representative images and quantitative data from functional assays (*n* = 5). **A**, **D** Transwell migration assay (scale bar = 200 μm). **B**, **E** Flow cytometric apoptosis analysis, and (**C**, **F**) Matrigel tube formation assay (scale bar = 100 μm). **G** CCK-8 proliferation assay. Data are presented as mean ± SD. *p < 0.05, **p < 0.01, ***p < 0.001, ****p < 0.0001, ns = nonsignificant

To determine whether the PI3K/AKT pathway mediates the functional enhancement by ECSW, we treated ECFCs with the PI3K inhibitor LY294002. Transwell migration assays showed that the enhanced migratory capacity of ECFCs induced by ECSW was suppressed by LY294002 (Fig. 3A, D). Similarly, the PI3K inhibitor reversed the pro-proliferative (Fig. 3G) and anti-apoptotic (Fig. 3B, E) effects of ECSW. Furthermore, in Matrigel tube formation assays, the enhanced angiogenic capacity of ECFCs after ECSW treatment was partially abolished by PI3K inhibition (Fig. 3C, F). Corroborating these findings, ECSW-induced VEGF-A secretion was also significantly attenuated by LY294002 (Supplementary Fig. 5E-F). Collectively, these results demonstrate that the PI3K/AKT pathway is central to the ECSW-induced enhancement of ECFC migration, proliferation, tube formation, and VEGF-A secretion, as well as the reduction in apoptosis.

###  ECSW preconditioning enhances ECFC homing and ameliorates acute cardiac injury post-MI

**Fig. 4:**
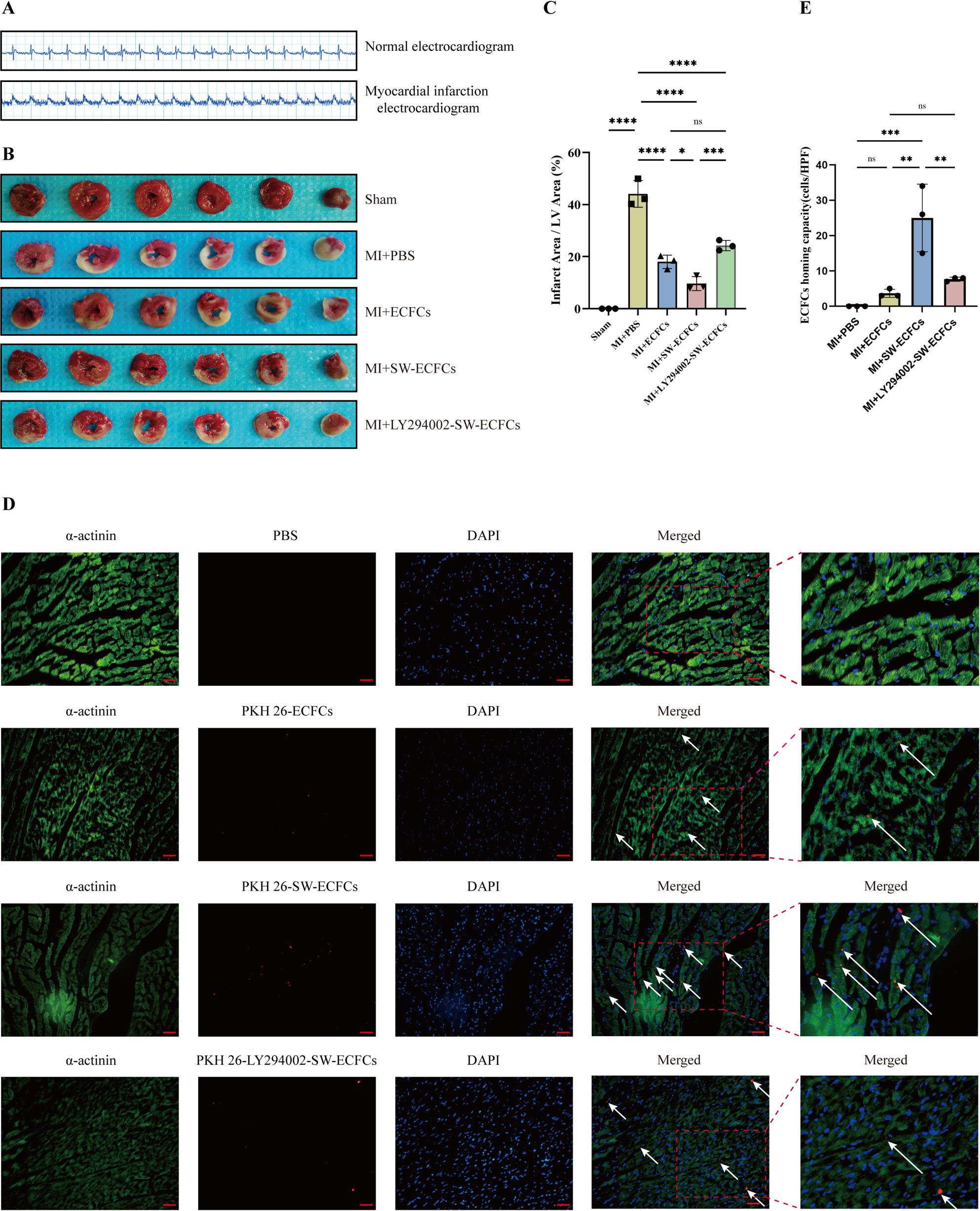
ECSW enhances ECFCs homing and ameliorates acute cardiac injury in a rat MI model. **A** Representative electrocardiogram traces from sham and MI model rats. **B**, **C** Representative TTC-stained heart sections and quantitative analysis of infarct size (*n* = 3). **D**, **E** Representative immunofluorescence images and quantification of PKH26-labeled ECFCs homing to the infarct border zone (*n* = 3; scale bar = 100 μm). Data are presented as mean ± SD. *p < 0.05, **p < 0.01, ***p < 0.001, ****p < 0.0001, ns = nonsignificant

 Following left anterior descending (LAD) coronary artery ligation, myocardial infarction (MI) was confirmed by ST-segment elevation on electrocardiogram (ECG) (Fig. 4A). Serum levels of creatine kinase (CK), CK-MB, and lactate dehydrogenase (LDH) were significantly elevated in all MI groups compared to the Sham group at 48 h, indicating similar levels of initial ischemic injury. Notably a trend toward lower enzyme levels was observed in the MI + SW-ECFCs group, although differences between treatment groups were not statistically significant (Supplementary Fig. 6 and Supplementary data Tables 2, 3 and 4). To investigate the mechanism of enhanced repair, we tracked the homing of PKH-26-labeled ECFCs at 6 h post-injection. Immunofluorescence analysis revealed significantly enhanced homing of cells to the infarct border zone in the MI + SW-ECFCs group compared to the MI + ECFCs group. This enhancement was abolished by PI3K inhibition (MI + LY294002-SW-ECFCs group), indicating that ECSW-facilitated homing depends on the PI3K/AKT pathway (Fig. 4D, E). Consistent with improved homing, TTC staining showed that treatment with SW-ECFCs significantly reduced the myocardial infarct size, a therapeutic effect that was markedly attenuated by LY294002 pretreatment (Fig. 4B, C). Collectively, these data confirm that ECSW preconditioning augments the therapeutic efficacy of ECFCs by enhancing their PI3K/AKT-dependent homing to the ischemic myocardium.

### ECSW-mediated activationof ECFCs preserves cardiac function in post-MI rats via the PI3K/AKT signaling pathway

**Fig. 5:**
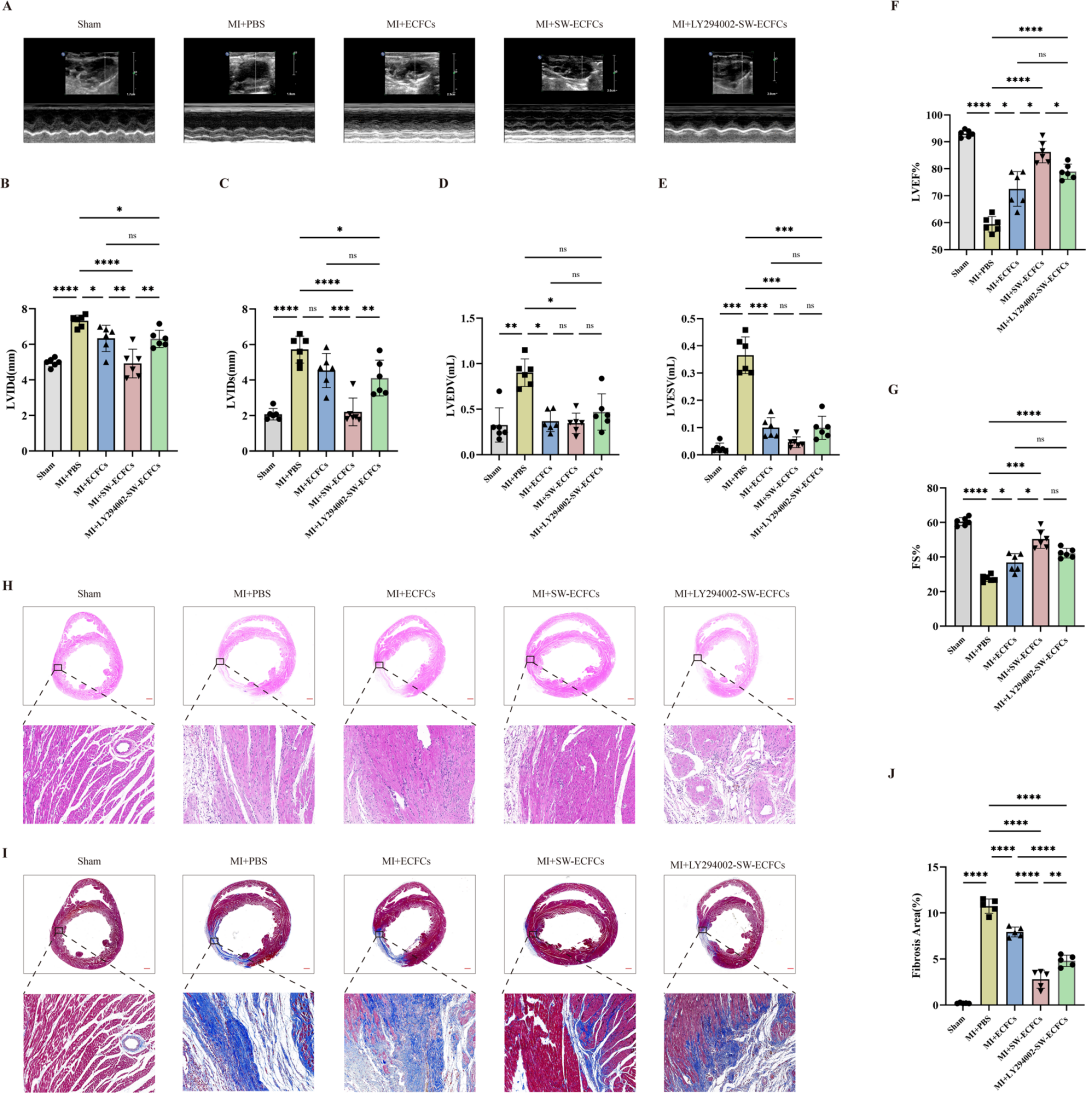
ECSW-activated ECFCs preserve cardiac function and attenuate remodeling post-MI via the PI3K/AKT pathway. **A**–**G** Representative echocardiographic images and quantitative analysis of cardiac function parameters (*n* = 6). **H**–**J** Representative H&E and Masson’s trichrome-stained heart sections and quantitative analysis of fibrosis (*n* = 5; scale bar = 1 mm). Data are presented as mean ± SD. *p < 0.05, **p < 0.01, ***p < 0.001, ****p < 0.0001, ns = nonsignificant

Transthoracic echocardiography performed 21 days post-MI revealed significant ventricular dilation (increased LVIDd, LVIDs , LVEDV, and LVESV) and impaired cardiac function (decreased LVEF and LVFS) in the MI + PBS group compared to the Sham group. Treatment with ECFCs mitigated these pathological alterations (Fig. 5A–G). Notably, the MI + SW-ECFCs group demonstrated a greater improvement in LVEF and LVFS, along with less ventricular dilation, compared to the MI + ECFCs group. In contrast, the benefits conferred by SW-ECFCs were partially abolished in the MI + LY294002-SW-ECFCs group, although cardiac parameters in this group remained superior to those of the MI + PBS group (Fig. 5A–G). Histological assessment via H&E and Masson’s trichrome staining corroborated the functional data. Myocardial damage was attenuated in the ECFCs-treated groups, as evidenced by significantly reduced inflammatory infiltration in the infarct border zone and smaller fibrotic areas compared to the MI + PBS group (Fig. 5H–J). ECSW-enhanced ECFCs yielded superior protective effects relative to untreated ECFCs. However, this protective effect was partially reversed by PI3K/AKT inhibition (MI + LY294002-SW-ECFCs group), which exhibited larger fibrotic areas compared to the MI + SW-ECFCs group (Fig. 5H–J). Collectively, these results confirm that ECSW preconditioning attenuates post-MI inflammatory responses and fibrotic remodeling by activating ECFCs via the PI3K/AKT signaling pathway.

### ECSW-mediated activation of ECFCs suppressespost-MI myocardial apoptosis via the PI3K/AKT signaling pathway

**Fig. 6:**
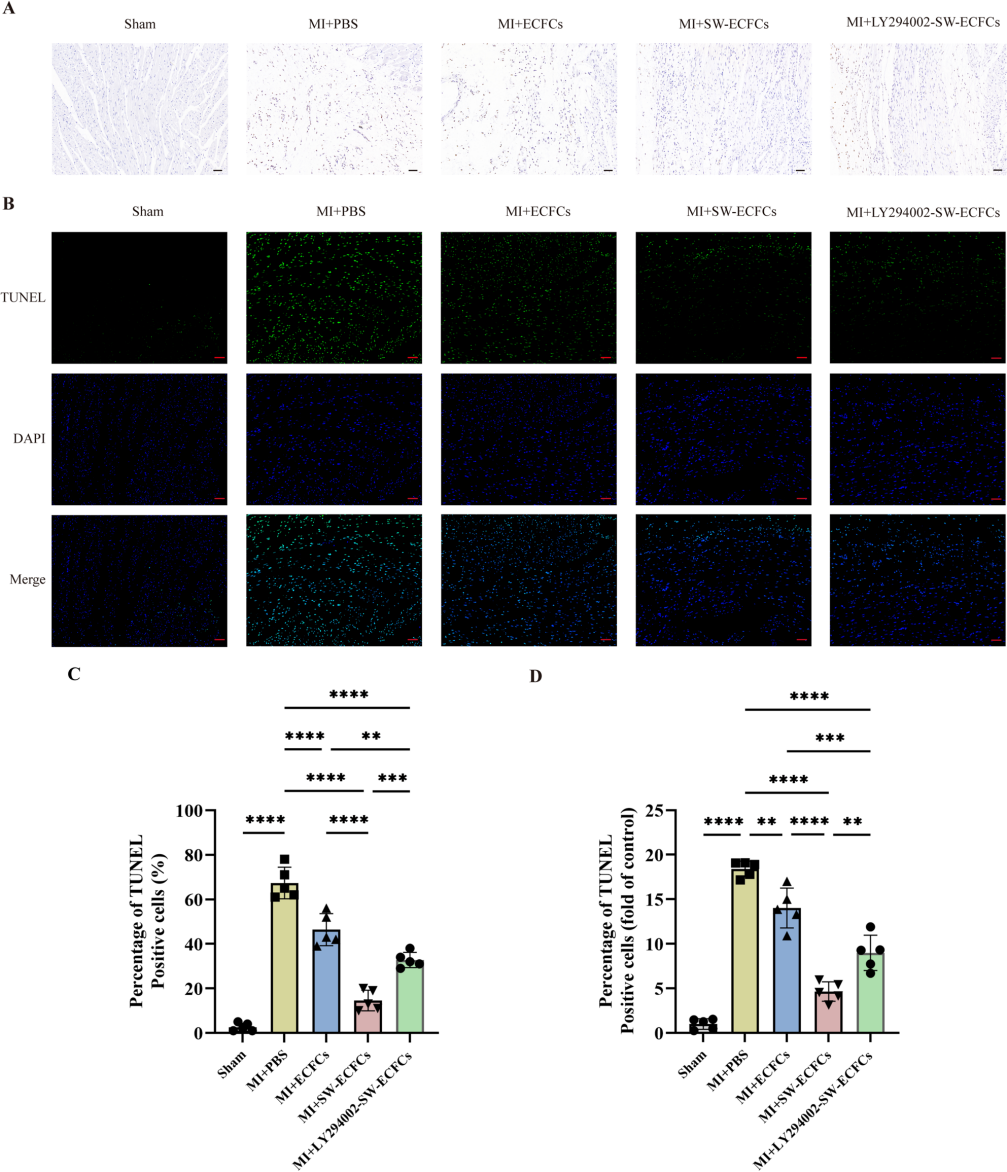
ECSW-activated ECFCs suppress myocardial apoptosis post-MI via the PI3K/AKT pathway. **A**–**D** Representative TUNEL staining images (IHC and IF) and quantitative analysis of apoptotic cells in the infarct border zone (*n* = 5; scale bar = 100 μm). Data are presented as mean ± SD. *p < 0.05, **p < 0.01, ***p < 0.001, ****p < 0.0001, ns = nonsignificant

To evaluate myocardial apoptosis post-MI, TUNEL immunohistochemistry and immunofluorescence staining were performed at 21 days post-MI. Compared to the MI + PBS group, both the MI + ECFCs and MI + SW-ECFCs groups showed significantly fewer apoptotic cells, with the most pronounced reduction in the MI + SW-ECFCs group (Fig. 6A–D). Notably, apoptosis in the MI + LY294002-SW-ECFCs group was higher than that in the MI + SW-ECFCs group but remained significantly lower than in the MI + PBS group (Fig. 6A–D).

### ECSW-mediated ECFCs activation promotes post-MI angiogenesis via the PI3K/AKT signaling pathway

**Fig. 7:**
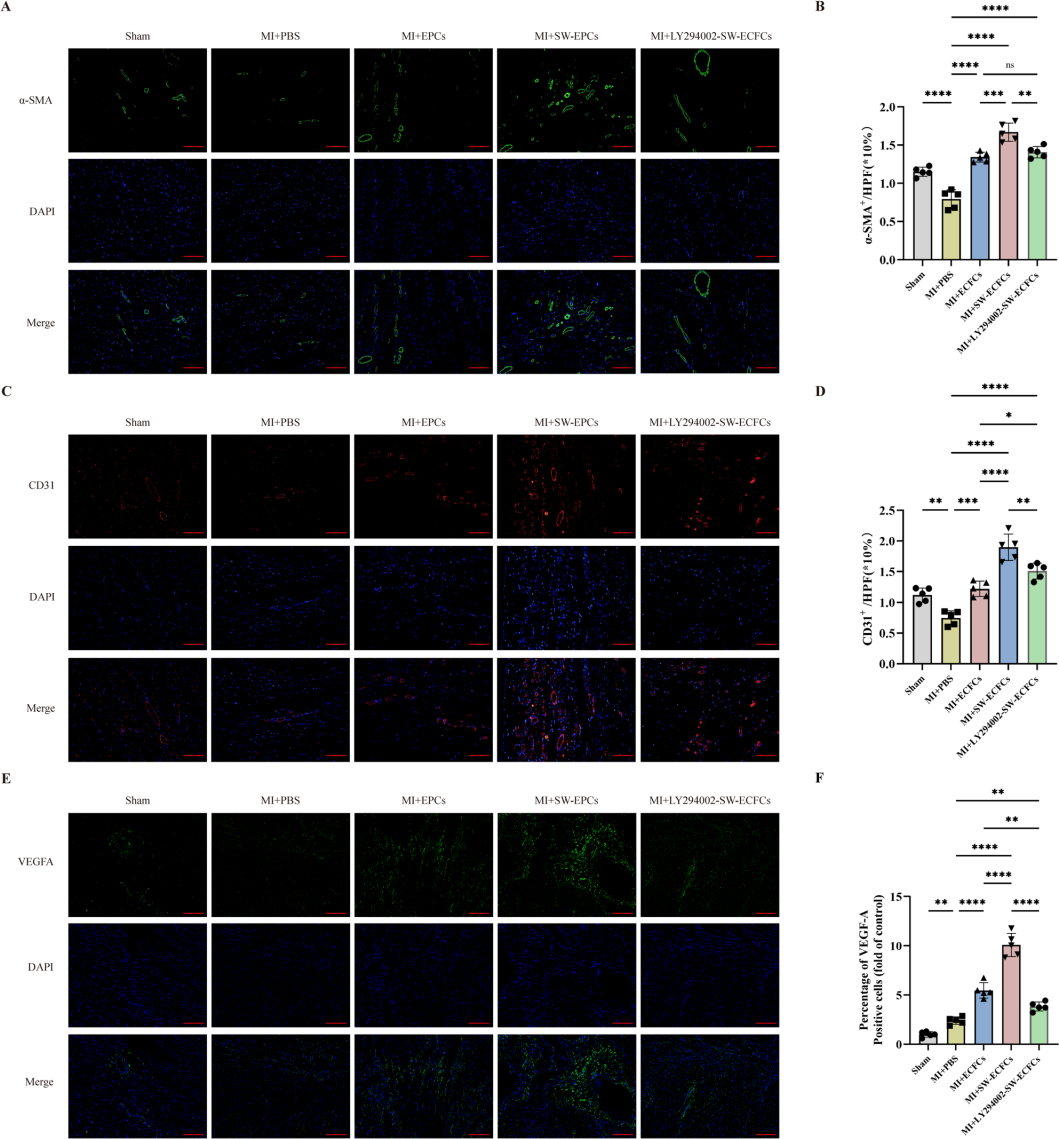
ECSW-activated ECFCs promote angiogenesis post-MI via the PI3K/AKT pathway. **A**–**F** Representative immunofluorescence images and quantitative analysis of vascular markers α-SMA, CD31, and VEGF-A in the infarct border zone (*n* = 5; scale bar = 50 μm). Data are presented as mean ± SD. *p < 0.05, **p < 0.01, ***p < 0.001, ****p < 0.0001, ns = nonsignificant

We investigated the angiogenic contribution of SW-ECFCs in post-MI rats. Expression of the vascular markers α‑smooth muscle actin (α‑SMA), CD31, and VEGF‑A in the infarct border zone was significantly increased in both the MI + ECFCs and MI + SW‑ECFCs groups compared to the MI + PBS group, with the highest levels observed in the MI + SW-ECFCs group (Fig. 7A–F). Levels of these markers in the MI + LY294002-SW-ECFCs group were lower than those in the MI + SW-ECFCs group but remained significantly higher than in the MI + PBS group (Fig. 7A–F). These results indicate that ECSW enhances ECFC-mediated post-MI angiogenesis in a PI3K/AKT-dependent manner .

### SW-ECFCs mediate cardioprotective effects by modulating the AKT/eNOS signaling pathway in a rat MI model

**Fig. 8:**
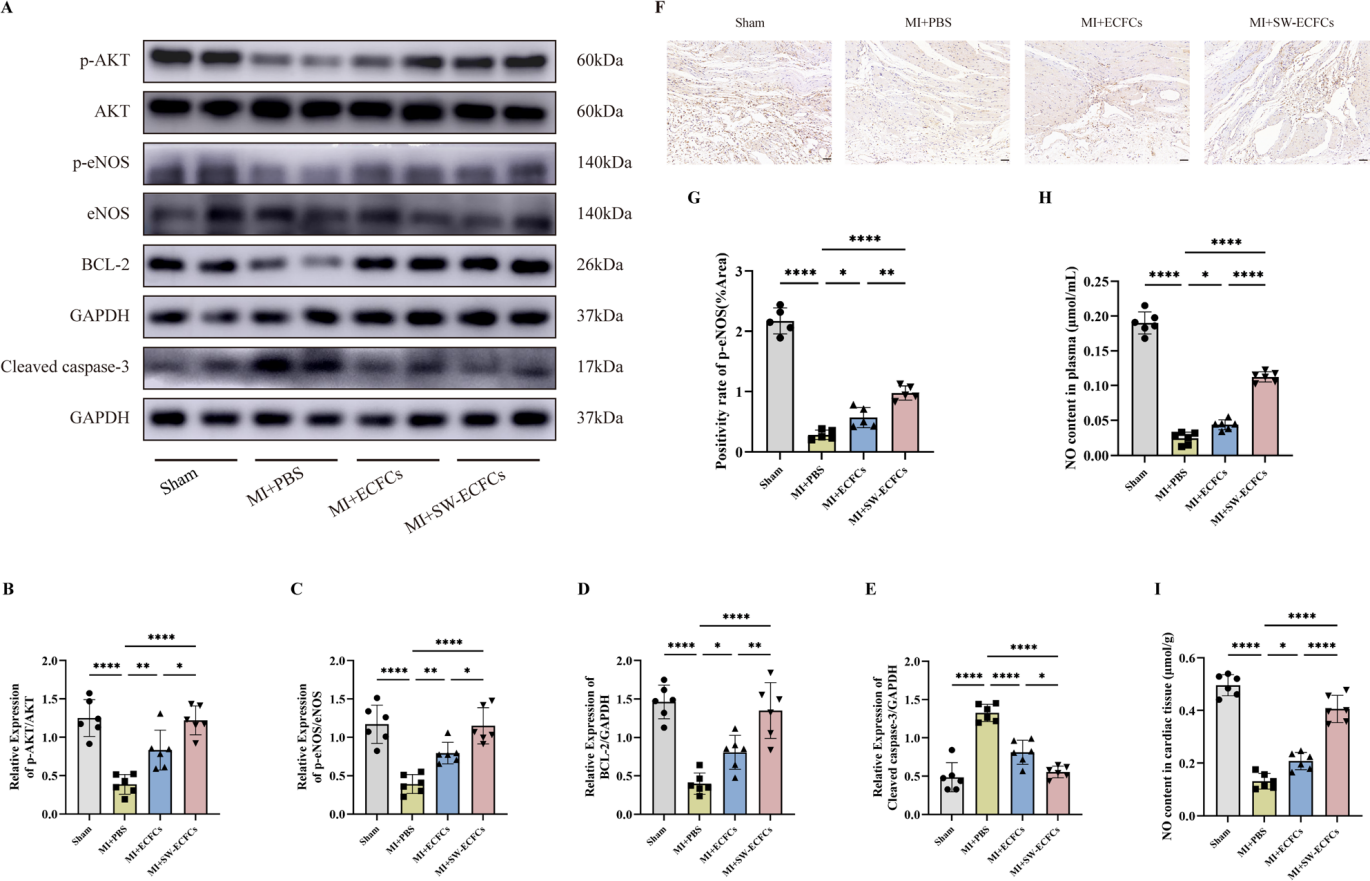
ECSW-activated ECFCs mediate cardioprotection by modulating the AKT/eNOS pathway in MI rats. **A**–**E** Western blot analysis and quantification of p-AKT, AKT, p-eNOS, eNOS, Bcl-2, and cleaved caspase-3 protein levels in cardiac tissue (*n* = 6). **F**, **G** Representative IHC images and quantitative analysis of p-eNOS expression (*n* = 5; scale bar = 100 μm). **H**, **I** Nitric oxide (NO) levels in myocardial tissue and plasma (*n* = 6). Data are presented as mean ± SD. Data are presented as mean ± SD. *p < 0.05, **p < 0.01, ***p < 0.001, ****p < 0.0001, ns = nonsignificant

**Fig. 9:**
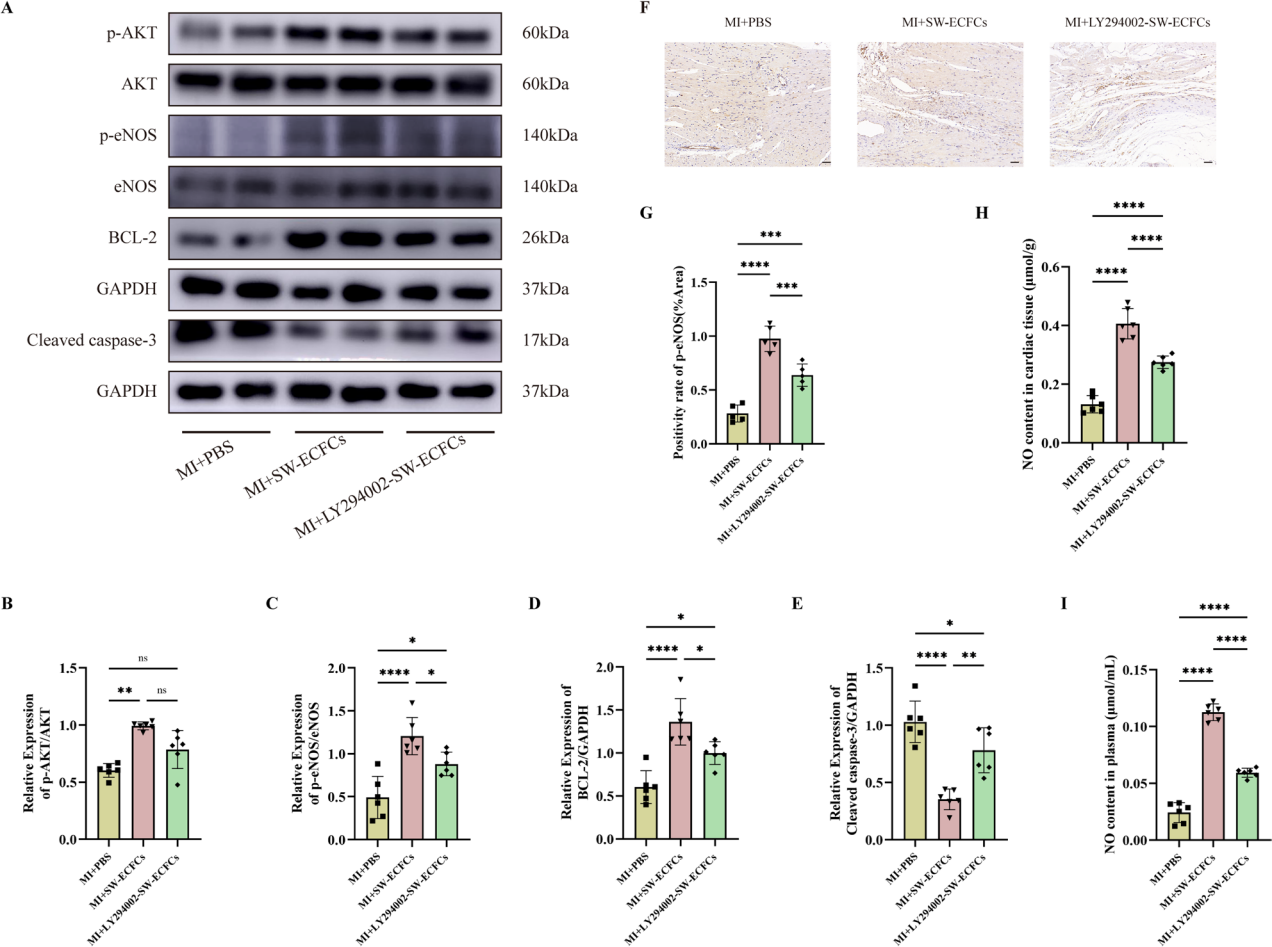
PI3K inhibition attenuates the cardioprotective effects of SW-ECFCs via the AKT/eNOS pathway. **A**–**E** Western blot analysis and quantification of p-AKT, AKT, p-eNOS, eNOS, Bcl-2, and cleaved caspase-3 in cardiac tissue from rats treated with LY294002-pretreated SW-ECFCs (*n* = 6). **F**, **G** Representative IHC images and quantitative analysis of p-eNOS expression (*n* = 5; scale bar = 100 μm). **H**, **I** Myocardial and plasma NO levels (*n* = 6). Data are presented as mean ± SD. *p < 0.05, **p < 0.01, ***p < 0.001, ****p < 0.0001, ns = nonsignificant

Given the crucial role of the PI3K/AKT/eNOS pathway in post-MI cardiac functional recovery and its involvement in mediating ECSW-induced ECFCs activation, we analyzed pathway alterations in MI rats treated with SW-ECFCs using Western blotting. As depicted in Fig. 8A–E, MI markedly reduced the levels of phosphorylated Akt (p-Akt), phosphorylated eNOS (p-eNOS), and Bcl-2, while increasing the level of cleaved caspase-3. Compared with PBS or ECFCs treatment, SW-ECFCs treatment significantly upregulated these pro-survival proteins (p-AKT, p-eNOS, Bcl-2) and downregulated cleaved caspase-3, without affecting the expression of total Akt or eNOS. Notably, LY294002-pretreated SW-ECFCs treatment attenuated these regulatory effects compared to SW-ECFCs treatment alone, though the pathway activation remained statistically significant relative to the PBS control (Figs. 9A–E). Consistent with these findings, immunohistochemical staining for p-eNOS and measurements of nitric oxide (NO) in myocardial tissue and plasma showed expression patterns that paralleled the Western blot results (Figs. 8F–I and 9F–I) .

**Fig. 10:**
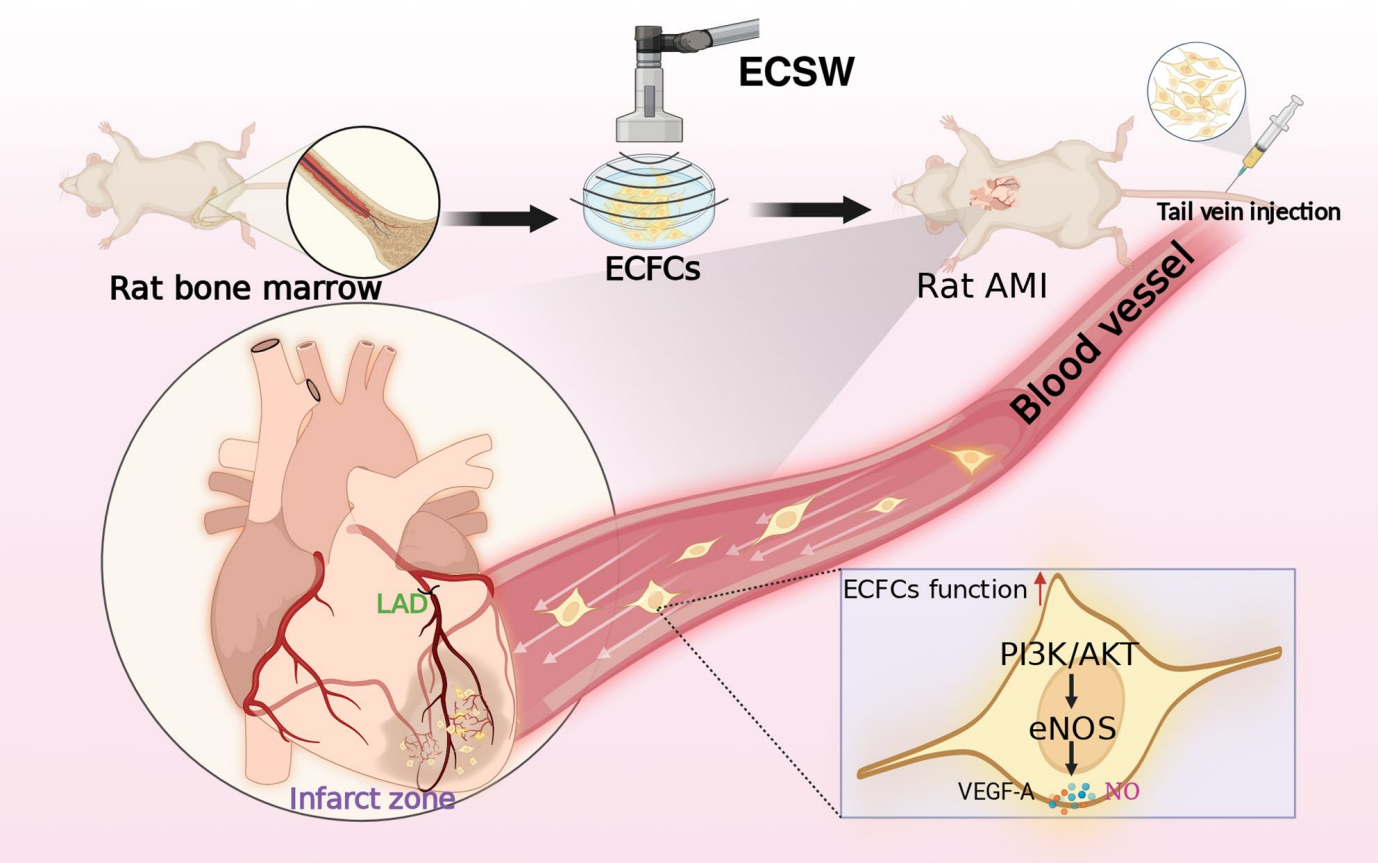
Attenuation of myocardial infarction by ECSW-primed ECFCs via the PI3K/Akt pathway This diagram illustrates the proposed mechanism by which ECSW preconditioning enhances the therapeutic efficacy of ECFCs against MI.It highlights the central role of the PI3K/Akt pathway, which was functionally validated in this study as the key mediator of the enhanced cellular functions

## Discussion

This study demonstrates that intravenous administration of extracorporeal cardiac shock wave-preconditioned endothelial colony-forming cells (SW-ECFCs) significantly improves cardiac function and promotes structural repair in a clinically relevant ApoE^-/-^ rat model of myocardial infarction. Our findings establish that ECSW preconditioning transforms ECFCs into potent therapeutic agents, primarily through activating the PI3K/AKT pathway . This activation augments the cells’ intrinsic functional capacities, promotes their homing to the ischemic myocardium, and orchestrates a multifaceted cardioprotective response.

Stem cell therapy represents a promising approach for treating myocardial infarction (MI) [27–29]. While early “endothelial progenitor cells” (EPCs) identified in 1997 [30] lacked true progenitor properties [31–33], definitive ECFCs capable of de novo vasculogenesis were later characterized [34]. Here, ECFCs isolated from ApoE^-/-^ rats displayed the classic phenotypic profile (CD31⁺/CD34⁺/CD146⁺/VEGFR2⁺, CD133⁺/VE‑cadherin⁺, CD14⁻/CD45⁻) [35, 36]. Notably, while wild-type ECFCs showed only a non-significant trend toward enhanced function compared to ApoE^-/-^ rat-derived ECFCs at baseline, ECSW preconditioning profoundly enhanced the functionality of ApoE^-/-^ ECFCs, confirming its strong regenerative potential. The use of the ApoE^-/-^ model was a deliberate choice to enhance clinical relevance, as MI in patients frequently occurs against a background of atherosclerotic cardiovascular disease, providing a more rigorous assessment of therapeutic potential.

ECFCs promote myocardial regeneration through direct endothelial differentiation and potent paracrine-mediated activation of endogenous repair mechanisms [37, 38]. While technical constraints prevented definitive tracking of donor ECFCs integration into vessels at 21 days, several observations strongly support a paracrine‑dominant mechanism. The widespread AKT/eNOS activation observed in host myocardium, which clearly contrasted with the sparse initial homing of donor cells, provides compelling evidence for this mode of action. This is consistent with reports that mechanical stimulation enhances paracrine secretion [39, 40]. This conclusion is further supported by our demonstration that ECSW enhances paracrine secretion, as evidenced by the PI3K-dependent increase in VEGF-A release from SW-ECFCs. Furthermore, the presence of isolated, bright PKH26^+^ cells suggests a quiescent donor population, aligning with the secretory phenotype of many transplanted progenitors and reinforcing their primary role as paracrine effectors.

The route and timing of cell delivery are pivotal for therapeutic efficacy. We employed intravenous infusion to circumvent the limitations of intracoronary and intramyocardial approaches, such as incomplete coverage and procedural invasiveness [22]. The modest homing efficiency observed shortly after MI may be attributed to the adverse inflammatory environment and myocardial edema [41, 42], which also underpins the reported superiority of delayed administration at 1 week post-MI [43, 44], consistent with our two-dose protocol. The recruited ECFCs, likely homing via collateral circulation and chemotaxis, were well-tolerated at a dose of 1 × 10⁶ cells [44], which effectively balanced benefit and microcirculatory safety.

A central finding of this study is the comprehensive validation of the PI3K/AKT pathway as a critical mediator of the therapeutic action SW-ECFCs. Our transcriptomic analysis initially identified several pathways, including PI3K/AKT and ERK cascades, as significantly altered following ECSW treatment. For subsequent functional validation, we selectively focused on the PI3K/AKT pathway based on its strong enrichment significance and its central role in regulating cell survival, growth, and angiogenesis—processes highly relevant to our therapeutic hypothesis. This focus was functionally confirmed by a series of in vitro assays, in which PI3K inhibition abolished the ECSW-induced enhancements in ECFCs migration, proliferation, tube formation, VEGF-A secretion, and resistance to apoptosis. Most importantly, this pathway dependency was rigorously validated in vivo. SW-ECFCs administration conferred significant benefits, including improved ventricular function, reduced infarct size, suppressed apoptosis, decreased fibrosis, and increased angiogenesis—effects consistent with observations in a porcine ischemic cardiomyopathy model [45]. Crucially, all these improvements were substantially attenuated when the cells were preconditioned with the PI3K inhibitor LY294002. This attenuation confirms that PI3K/AKT activation within donor ECFCs is essential for the observed therapeutic effect. The partial, rather than complete, reversal of benefits by PI3K inhibition suggests that other ECSW-activated pathways identified in our transcriptomic data, such as the ERK cascade, may contribute synergistically or in parallel to the overall enhanced cellular state and therapeutic outcome, warranting further investigation.

Mechanistically, the benefits of SW-ECFCs stem from sustained activation of the PI3K/AKT/eNOS axis in the host myocardium, as demonstrated at the 21-day endpoint by elevated phosphorylation of AKT/eNOS, nitric oxide levels, and Bcl-2 expression. Crucially, we found that this pathway directly regulates the secretion of pivotal paracrine factors, evidenced in vitro by the PI3K inhibitor-mediated abolition of ECSW-induced VEGF-A release. The PI3K/AKT axis further enhanced the initial homing of SW-ECFCs, which were recruited to the infarct border zone in significantly greater numbers than their LY294002-preconditioned counterparts. Most compellingly, PI3K inhibition in donor cells weeks before analysis attenuated both pathway activation and VEGF expression in the host myocardium, providing powerful and direct evidence for a donor-cell-dependent paracrine mechanism. Collectively, these findings bridge our prior in vitro work [18, 26] with conclusive in vivo validation, establishing PI3K/AKT activation as a primary—though likely not exclusive—mechanism through which ECSW preconditioning potentiates the therapeutic efficacy of ECFCs.

The therapeutic outcomes observed over the 21-day period should be interpreted in the context of the disease progression timeline. Our data delineate a clear mechanistic distinction: serum biomarkers at 48 h confirmed comparable initial ischemic injury across all groups, and the absence of a significant reduction in the MI + SW-ECFC group indicates that the therapy’s mechanism is not centered on acute cytoprotection. Rather, the functional and structural improvements evident by day 21 are attributable to interventions in the subsequent reparative phase. SW-ECFCs appear to operate within this critical window, enhancing angiogenesis, constraining maladaptive remodeling, and fostering a pro-survival microenvironment to facilitate recovery.

This study has several limitations. First, the reliance on pharmacological inhibition, while supportive of PI3K/AKT involvement, necessitates genetic validation for definitive pathway specificity. Second, the 21-day endpoint precludes assessment of long-term therapeutic sustainability and obscures dynamic pathway activity. Third, the sole use of ECFCs from ApoE^-/-^ rats, in the absence of an isogenic wild-type control, complicates the separation of the ECSW-specific effect from the background of a dyslipidemic microenvironment. Fourth, the technical limitations of PKH-26 tracing prevented a definitive analysis of the long-term fate and proliferative capacity of ECFCs. Finally, although our focused transcriptomic analysis identified PI3K/AKT as a principal pathway, future unbiased global transcriptomic and proteomic profiling will be essential to comprehensively delineate the full spectrum of signaling networks activated by ECSW. Corresponding future directions should include: (i) utilizing large-animal models with extended follow-up; (ii) implementing advanced multimodal cell tracking technologies; (iii) conducting comprehensive multi-omics analyses to map the signaling landscape; and (iv) applying genetic tools for definitive pathway validation and investigating lineage commitment.

## Conclusion

This study reveals that preconditioning ECFCs with ECSW significantly enhances their therapeutic efficacy for myocardial infarction, leading to improved cardiac function and structural repair. These benefits are mediated primarily through activation of the PI3K/AKT signaling pathway, which augments cell homing, paracrine activity, and survival. This work offers a novel and promising strategy for cardiac regeneration.

## Supplementary Information

Below is the link to the electronic supplementary material.

Supplementary Material 1

Supplementary Material 2

## Data Availability

The RNA-seq datasets generated during this study are publicly available in the NCBI Sequence Read Archive (SRA) under BioProject accession number PRJNA1392865. All other relevant data (e.g., experimental measurements, statistical source data, and uncropped blots) are provided in the Supplementary Information or are available from the corresponding author upon reasonable request.

## Abbreviations

ECSW: Extracorporeal cardiac shock waves
ECFCs: Endothelial colony-forming cells
MI: Myocardial infarction
SW-ECFCs: ECSW-preconditioned ECFCs
LY-SW-ECFCs: LY294002-pretreated SW-ECFCs
TTC: Triphenyl tetrazolium chloride
NO: Nitric oxide
AMI: Acute myocardial infarction
PCI: Percutaneous coronary intervention
CABG: Coronary artery bypass grafting
EPCs: Endothelial progenitor cells
BMCs: Bone marrow-derived mononuclear cells
CHD: Coronary heart disease
EGM-2 MV: Endothelial growth medium-2 microvascular
LAD: Left anterior descending
CK: Creatine kinase
LDH: Lactate dehydrogenase
LVIDd: Left ventricular end-diastolic diameter
LVIDs: Left ventricular end-systolic diameter
LVEF: Left ventricular ejection fraction
LVEDV: Left ventricular end-diastolic volume
LVESV: Left ventricular end-systolic volume
LVF: Left ventricular fractional shortening
