# Supplementary material for: Extracorporeal cardiac shock wave stimulation enhances the therapeutic efficacy of intravenously delivered endothelial colony-forming cells via PI3K/AKT signaling in a rat myocardial infarction model
Source: Stem Cell Res Ther. 2026 Feb 1;17:91. doi: 10.1186/s13287-026-04913-w (PMC12952025; doi:10.1186/s13287-026-04913-w)
Supplement: Supplementary file 1 — Supplementary Material 1 [file 13287_2026_4913_MOESM1_ESM.docx]

Figure11

AKT/p-AKT


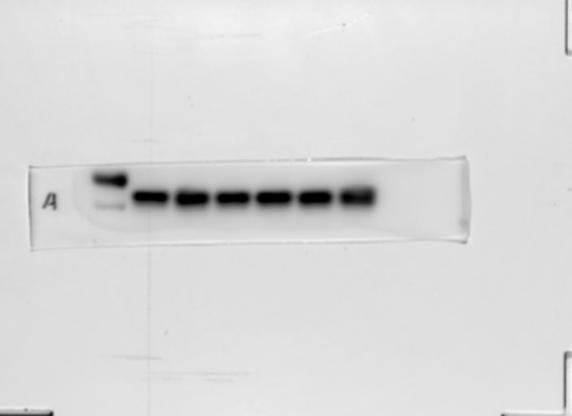

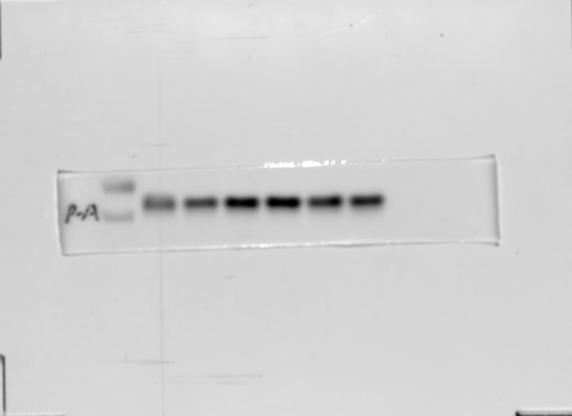


AKT-used p-AKT-used


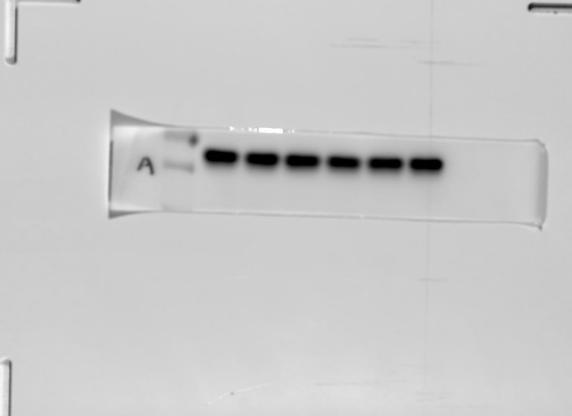

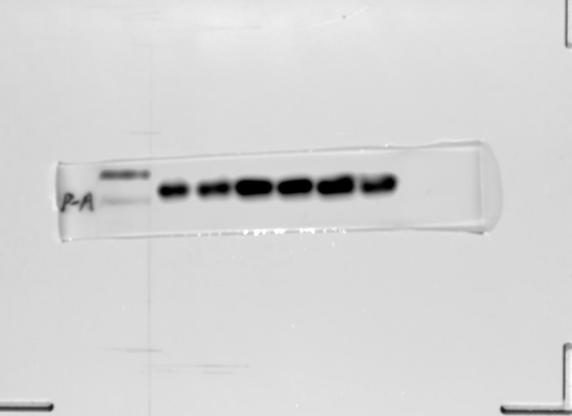


AKT p-AKT


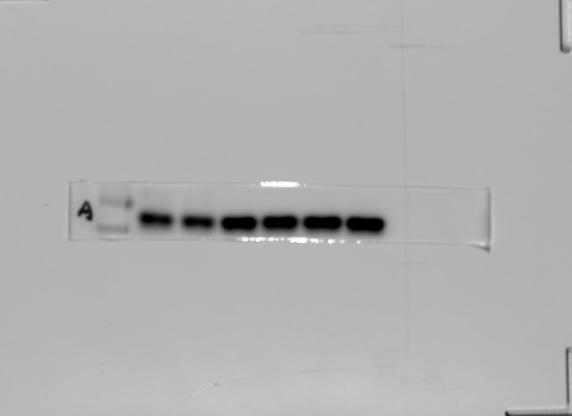

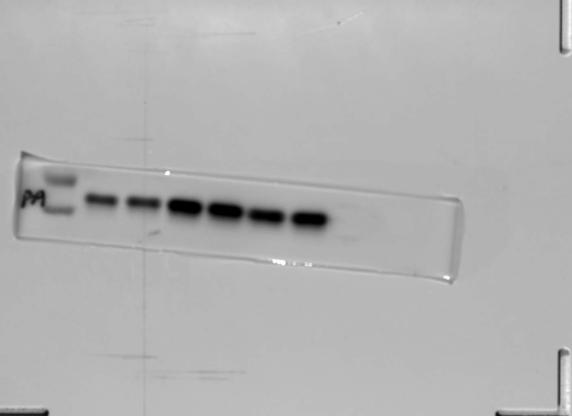


AKT p-AKT

eNOS/p-eNOS


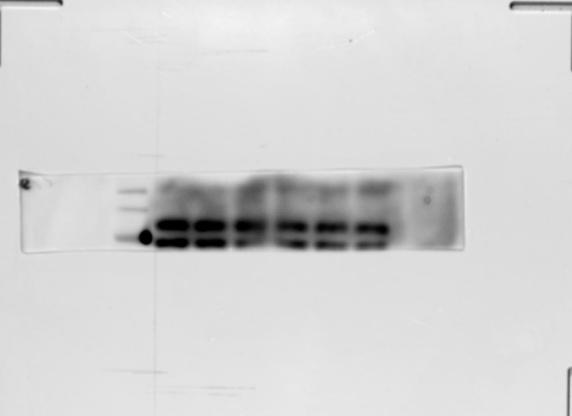

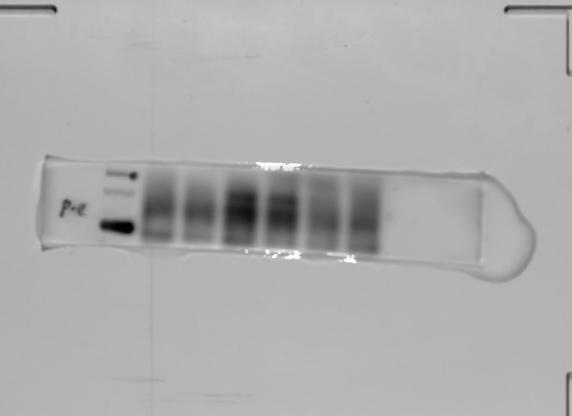


eNOS p-eNOS


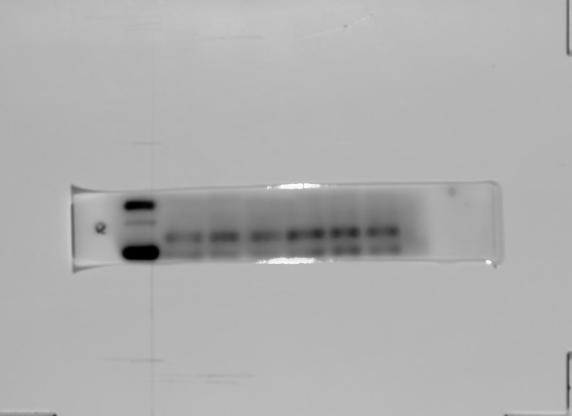

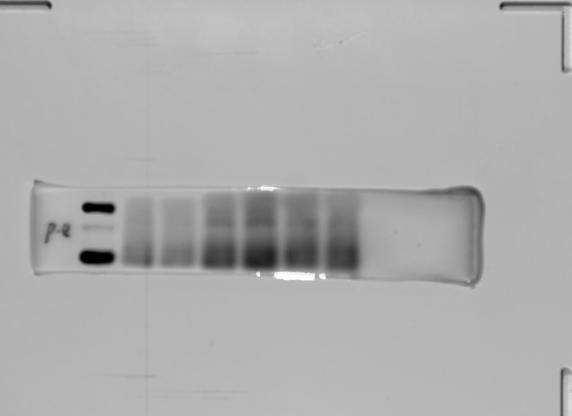


eNOS-used p-eNOS-used


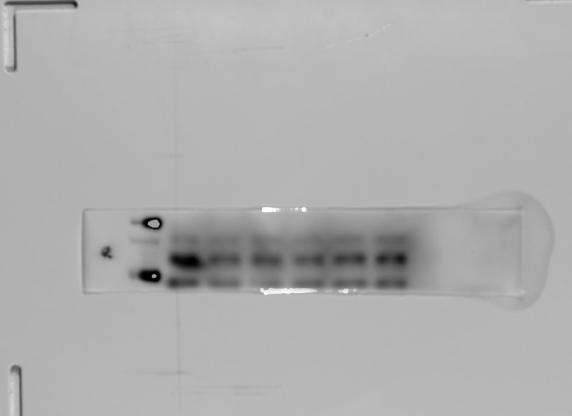

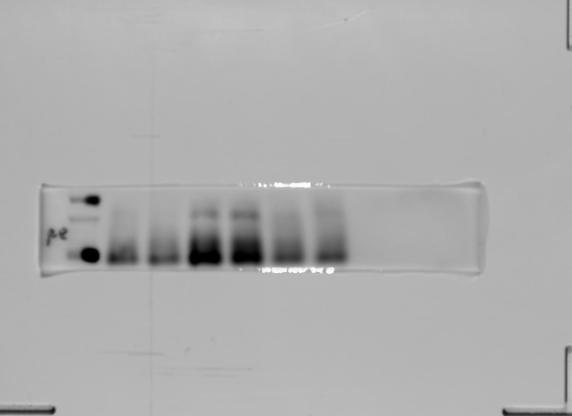


eNOS p-eNOS

BCL-2/GAPDH


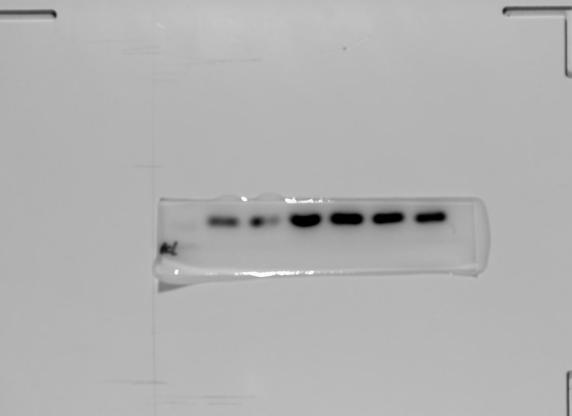

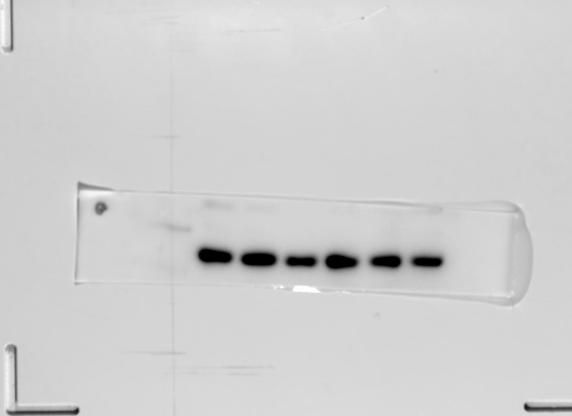


Bcl-2-used GAPDH-used


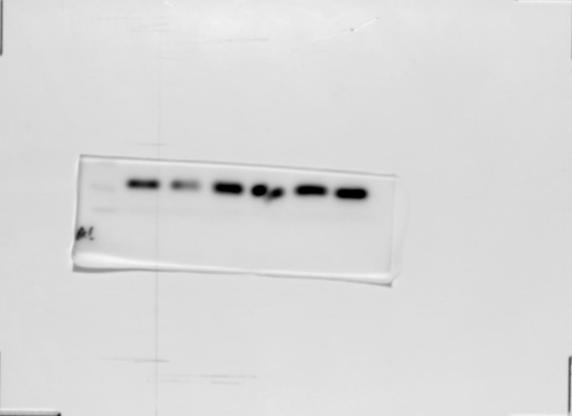

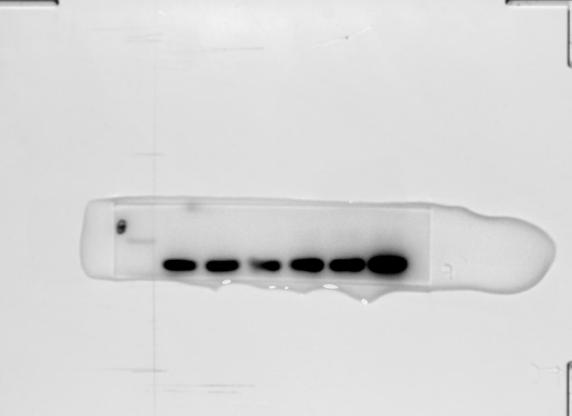


Bcl-2 GAPDH


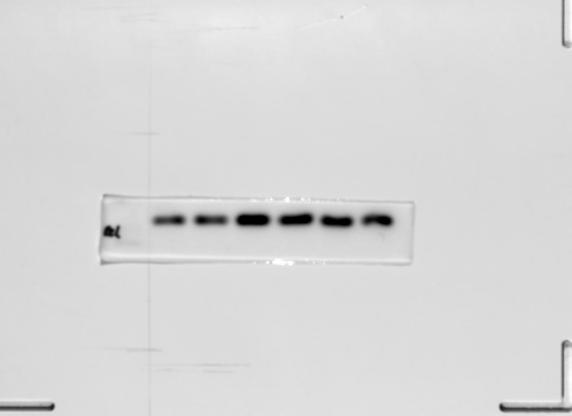

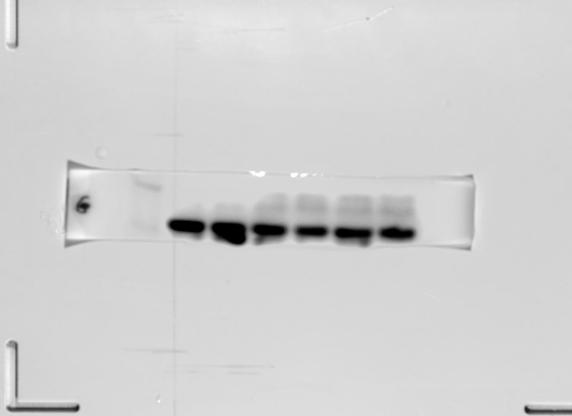


Bcl-2 GAPDH

cleaved-caspase-3/GAPDH


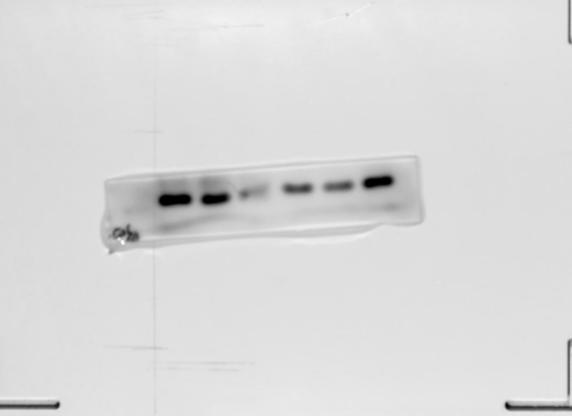

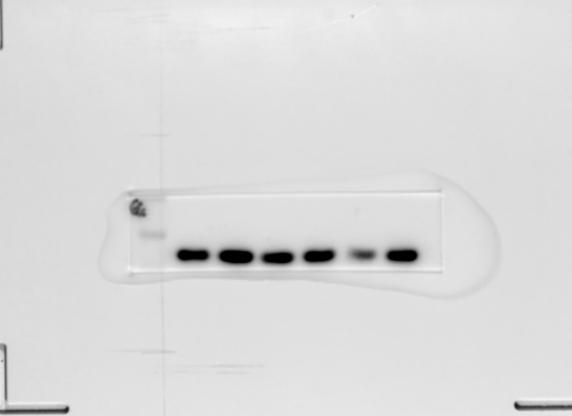


cleaved-caspase-3 GAPDH


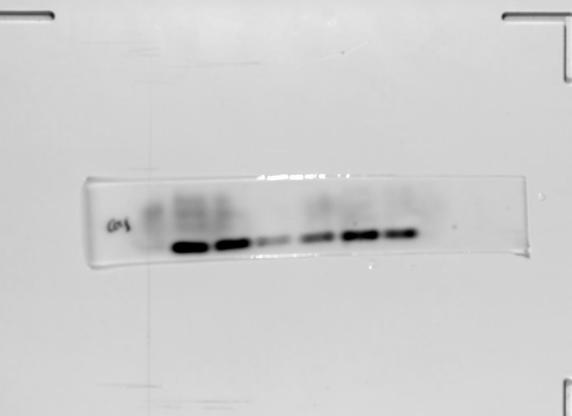

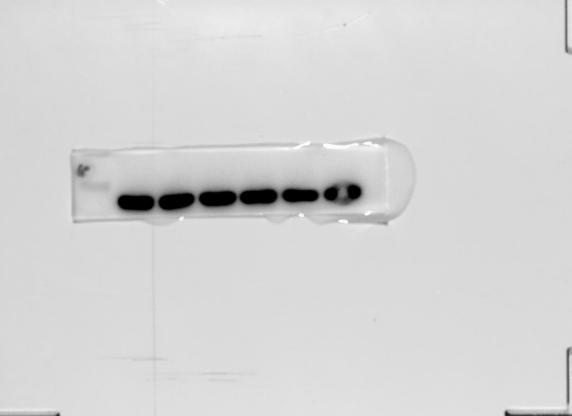


cleaved-caspase-3 GAPDH


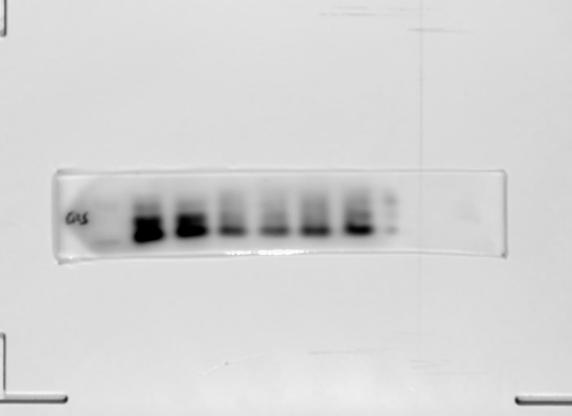

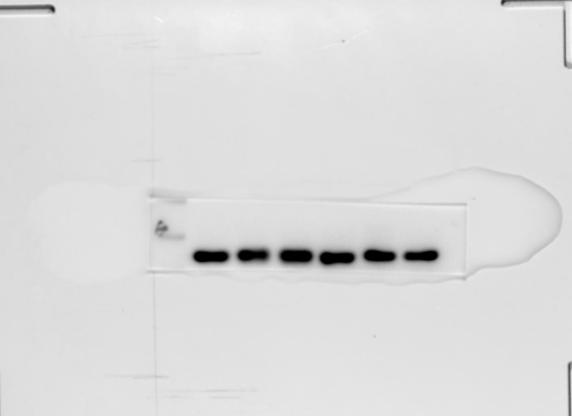


cleaved-caspase-3-used GAPDH-used

Figure10

p-AKT/AKT


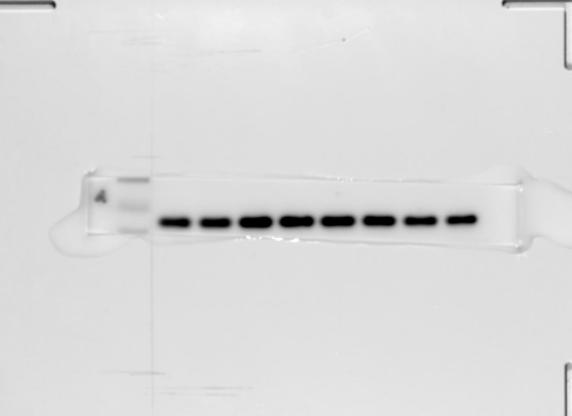

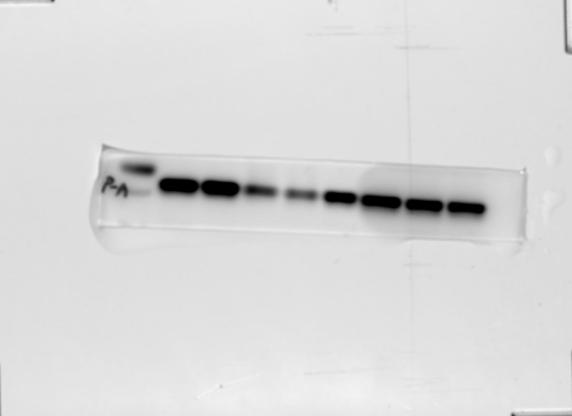


AKT p-AKT


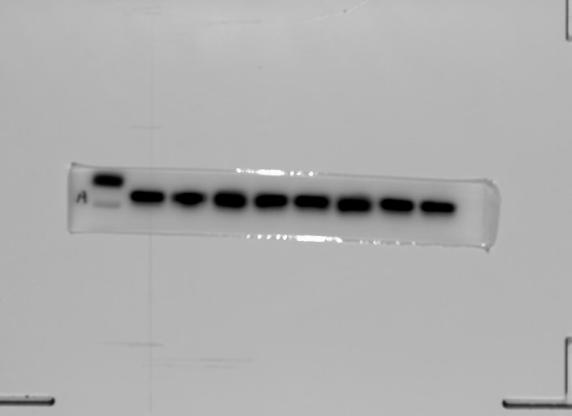

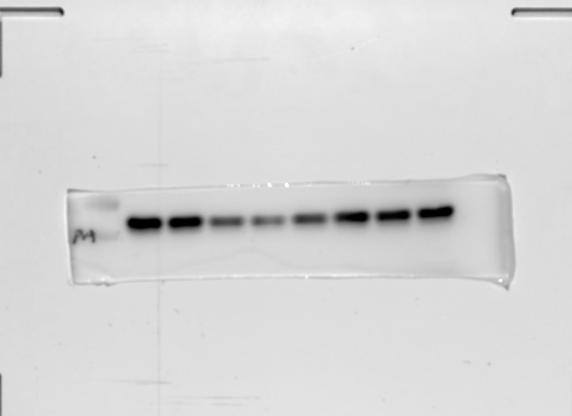


AKT-used p-AKT-used


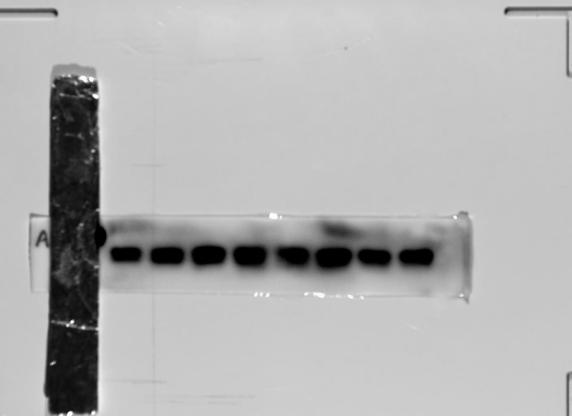

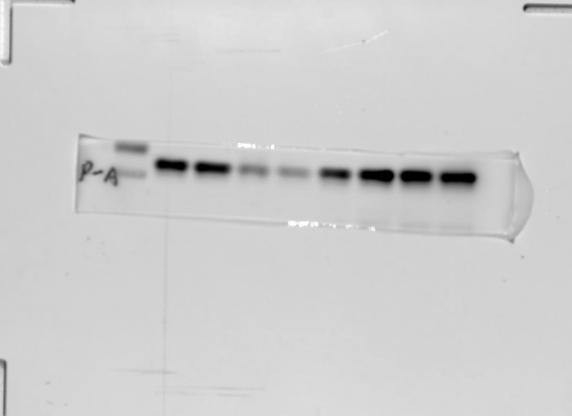


AKT p-AKT

p-eNOS/eNOS


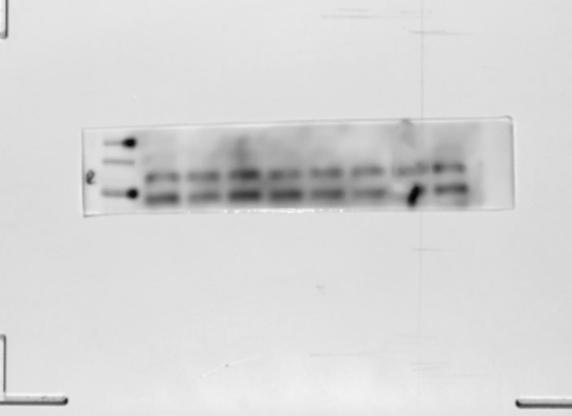

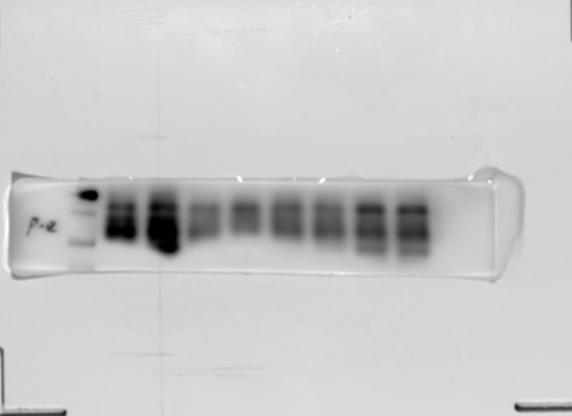


eNOS p-eNOS


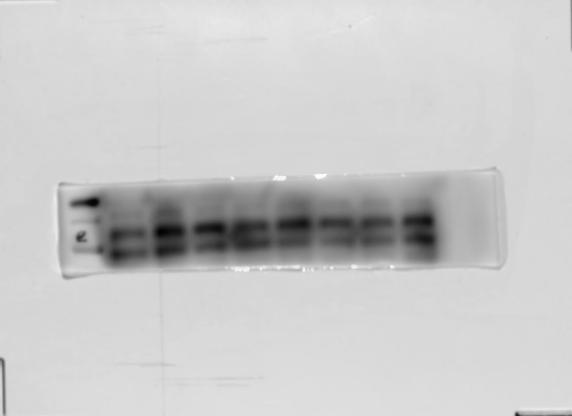

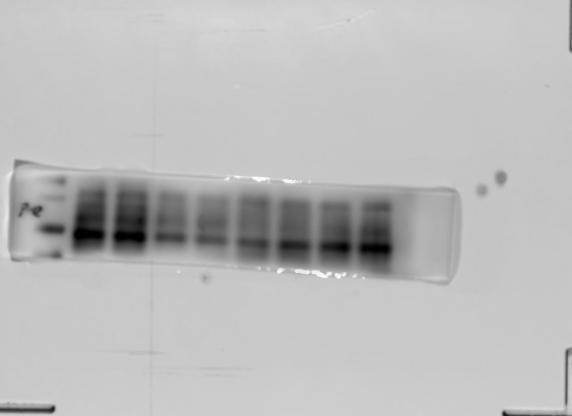


eNOS-used p-eNOS-used


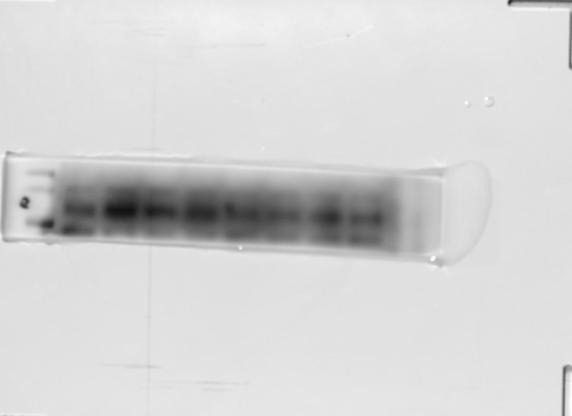

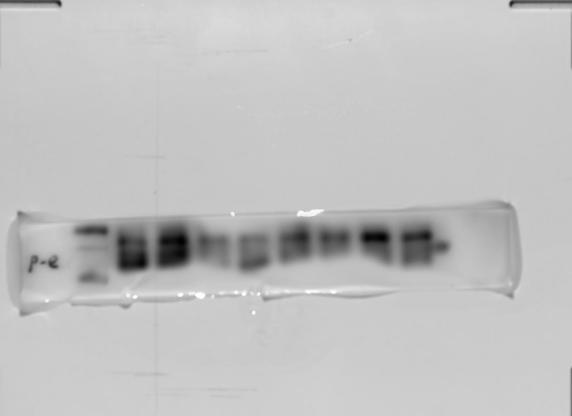


eNOS p-eNOS

BCL-2/GAPDH


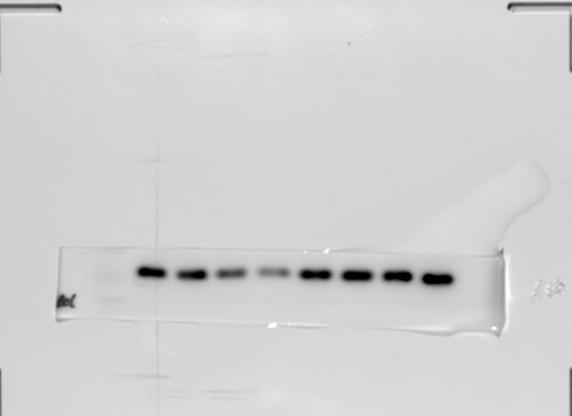

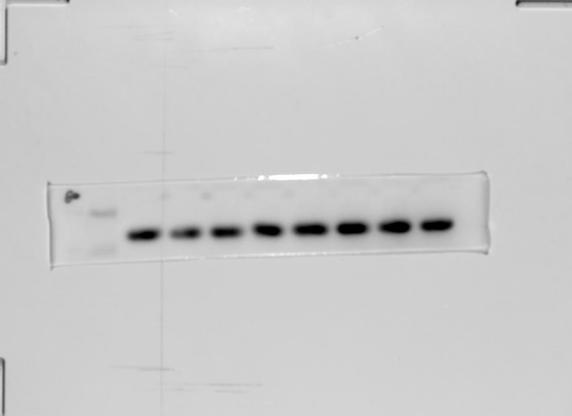


Bcl-2-used GAPDH-used


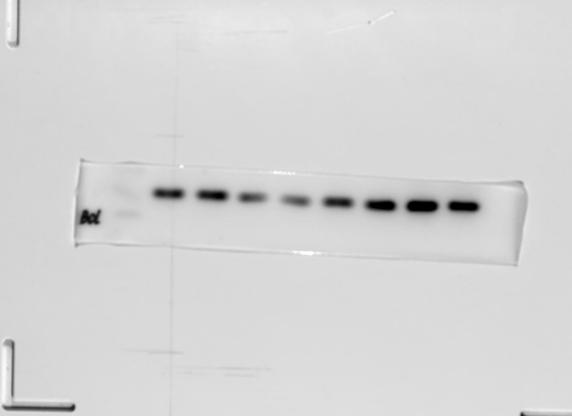

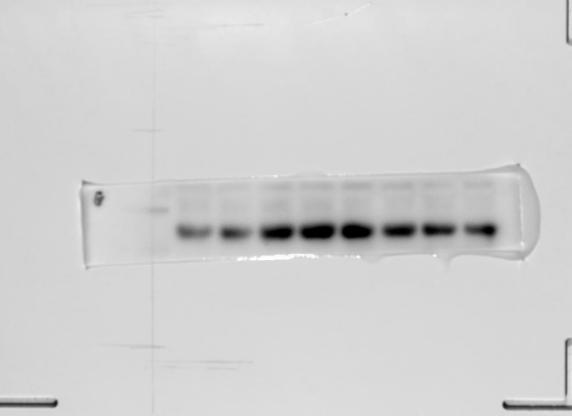


Bcl-2 GAPDH


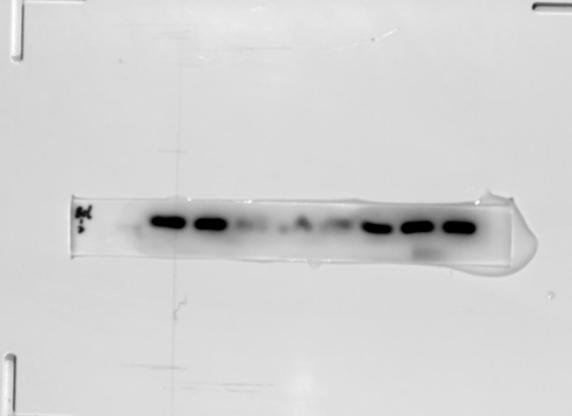

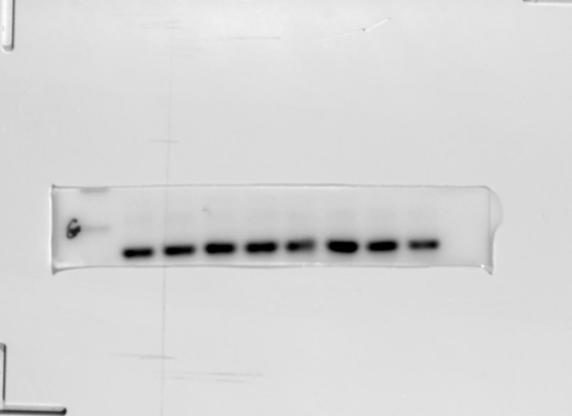


Bcl-2 GAPDH

cleaved-caspase-3/GAPDH


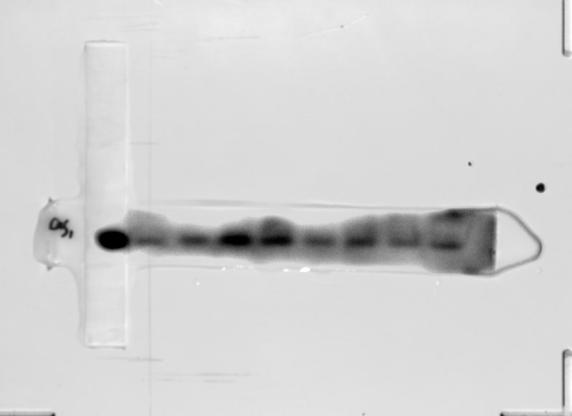

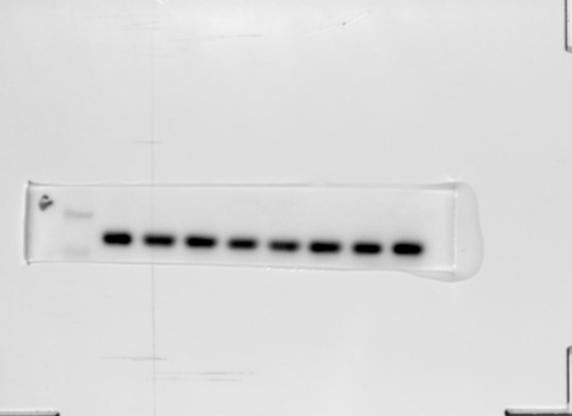


cleaved-caspase-3-used GAPDH-used


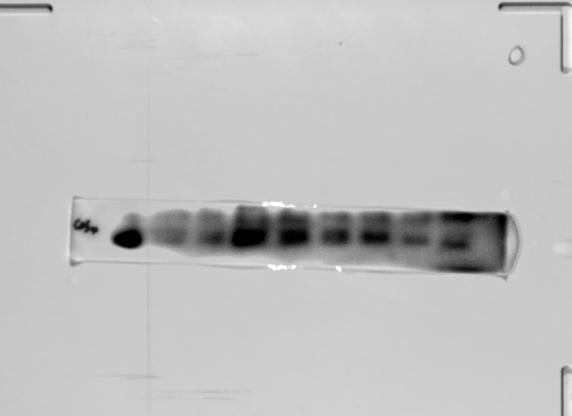

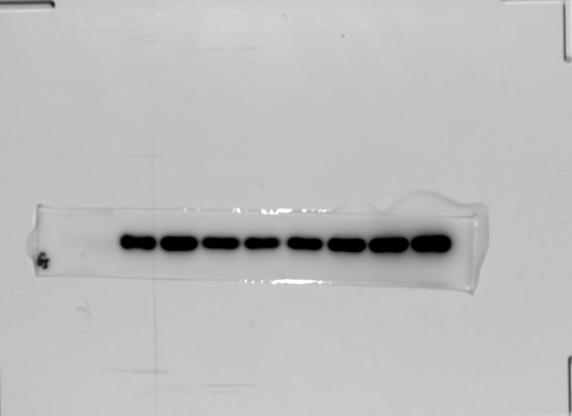


cleaved-caspase-3 GAPDH


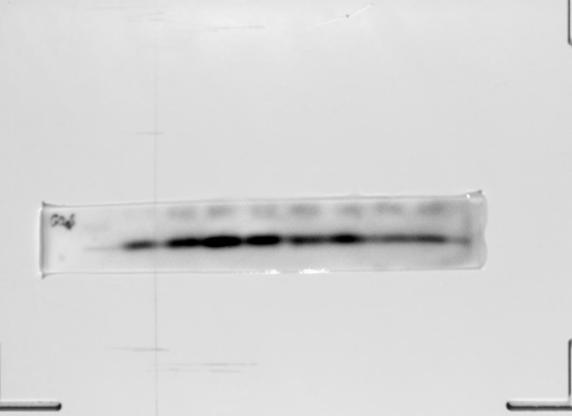

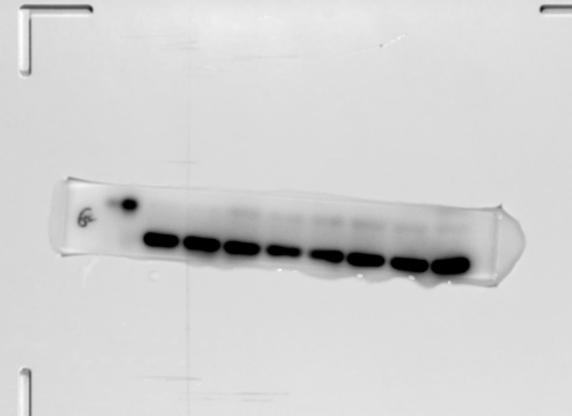


cleaved-caspase-3 GAPDH
