# Supplementary material for: Extracorporeal cardiac shock wave stimulation enhances the therapeutic efficacy of intravenously delivered endothelial colony-forming cells via PI3K/AKT signaling in a rat myocardial infarction model
Source: Stem Cell Res Ther. 2026 Feb 1;17:91. doi: 10.1186/s13287-026-04913-w (PMC12952025; doi:10.1186/s13287-026-04913-w)
Supplement: Supplementary file 2 — Supplementary Material 2 [file 13287_2026_4913_MOESM2_ESM.docx]

Supplementary data tables

Table S1. ECSW regulates the migration, apoptosis, tube formation, and proliferative capacities of ECFCs via the PI3K/AKT signaling pathway.

| Parameters | Control | ECSW | LY294002 | ECSW+LY294002 |
| --- | --- | --- | --- | --- |
| Total length of tubes | 1.000 ± 0.03046 | 1.561 ±0.03739 | 0.7440 ± 0.06745 | 1.237 ± 0.04685 |
| Total length of tubes (95% CIs) | [0.9622, 1.038] | [1.514, 1.607] | [0.6602, 0.8277] | [1.178, 1.295] |
| Cell migration | 312.4±21.14 | 499.6 ± 21.48 | 194.4 ± 19.46 | 387.6 ±21.71 |
| Cell migration (95% CIs) | [286.2, 338.6] | [472.9, 526.3] | [170.2, 218.6] | [360.6, 414.6] |
| Apoptosis(%) | 10.98±1.134 | 7.274±0.8242 | 18.83±0.8232 | 13.92±0.7106 |
| Apoptosis(95% CIs) | [7.318, 13.68] | [6.251, 8.297] | [17.80, 19.85] | [13.04, 14.80] |
| Cells proliferative | 0.5270±0.03048 | 0.8595±0.07022 | 0.2189±0.01203 | 0.4768±0.05330 |
| Cells proliferative(95% CIs) | [0.4892, 0.5648] | [0.7723, 0.9467] | [0.2039, 0.2338] | [0.4106, 0.5429] |

## Table S2. Serum creatine kinase (CK) levels across experimental groups.

| 项目名称 | 项目全称 | 原理方法 | | 方法类型 | | 主次波长 | | |
| --- | --- | --- | --- | --- | --- | --- | --- | --- |
|  |  |  |  |  |  |  |  |  |
| CK | 肌酸激酶 | 磷酸肌酸底物法 | | 速率法 | | 340/405 | | |
| 单位 | 测量时间(S) | 测试用量(µl) | | | | 测试样本量(µl) | | |
|  |  | R1 | | R2 | | 标准 | | |
| U/L | 120-300 | 200 | | 50 | | 10 | | |
| 注：编号标红表示样本微溶血，标黄表示样本颜色乳糜。 | | |  | |  | |  |  |
| 样本编号 | 项目 | 结果 | | 单位 | | | |  |
| MI+SW-ECFCs1 | CK | 2085.136 | | U/L | | | |  |
| MI+SW-ECFCs2 | CK | 1964.8 | | U/L | | | |  |
| MI+SW-ECFCs3 | CK | 1841.321 | | U/L | | | |  |
| MI+SW-ECFCs4 | CK | 2127.583 | | U/L | | | |  |
| MI+SW-ECFCs5 | CK | 2279.843 | | U/L | | | |  |
| MI+ECFCs1 | CK | 1982.597 | | U/L | | | |  |
| MI+ECFCs2 | CK | 2232.024 | | U/L | | | |  |
| MI+ECFCs3 | CK | 2019.871 | | U/L | | | |  |
| MI+ECFCs4 | CK | 2171.334 | | U/L | | | |  |
| MI+ECFCs5 | CK | 2208.891 | | U/L | | | |  |
| MI-1 | CK | 2080.573 | | U/L | | | |  |
| MI-2 | CK | 2143.939 | | U/L | | | |  |
| MI-3 | CK | 2294.9 | | U/L | | | |  |
| MI-4 | CK | 2174.484 | | U/L | | | |  |
| MI-5 | CK | 2384.362 | | U/L | | | |  |
| MI+LY-SW-ECFCs1 | CK | 1987.967 | | U/L | | | |  |
| MI+LY-SW-ECFCs2 | CK | 2064.386 | | U/L | | | |  |
| MI+LY-SW-ECFCs3 | CK | 2195.144 | | U/L | | | |  |
| MI+LY-SW-ECFCs4 | CK | 2272.326 | | U/L | | | |  |
| MI+LY-SW-ECFCs5 | CK | 1966.859 | | U/L | | | |  |
| sham5 | CK | 1125.641 | | U/L | | | |  |
| sham4 | CK | 936.267 | | U/L | | | |  |
| sham3 | CK | 1041.188 | | U/L | | | |  |
| sham2 | CK | 1102.886 | | U/L | | | |  |
| sham1 | CK | 841.807 | | U/L | | | |  |

## Table S3. Serum creatine kinase isoenzyme (CK-MB) levels across experimental groups.

| 项目名称 | 项目全称 | 原理方法 | 方法类型 | 主次波长 | |
| --- | --- | --- | --- | --- | --- |
|  |  |  |  |  |  |
| CK-MB | 肌酸激酶MB同工酶 | 免疫抑制法 | 速率法 | 340/405 | |
| 单位 | 测量时间(S) | 测试用量(µl) | | 测试样本量(µl) | |
|  |  | R1 | R2 | 标准 | |
| U/L | 120-260 | 240 | 60 | 12 | |
| 样本编号 | 项目 | 结果 | 单位 | |  |
| MI+SW-ECFCs1 | CK-MB | 2062.753 | U/L | |  |
| MI+SW-ECFCs2 | CK-MB | 2113.629 | U/L | |  |
| MI+SW-ECFCs3 | CK-MB | 1974.958 | U/L | |  |
| MI+SW-ECFCs4 | CK-MB | 2200.255 | U/L | |  |
| MI+SW-ECFCs5 | CK-MB | 1863.719 | U/L | |  |
| MI+ECFCs1 | CK-MB | 1903.493 | U/L | |  |
| MI+ECFCs2 | CK-MB | 2287.264 | U/L | |  |
| MI+ECFCs3 | CK-MB | 1981.78 | U/L | |  |
| MI+ECFCs4 | CK-MB | 2170.465 | U/L | |  |
| MI+ECFCs5 | CK-MB | 2048.39 | U/L | |  |
| MI-1 | CK-MB | 2213.241 | U/L | |  |
| MI-2 | CK-MB | 2116.9 | U/L | |  |
| MI-3 | CK-MB | 2095.197 | U/L | |  |
| MI-4 | CK-MB | 1924.02 | U/L | |  |
| MI-5 | CK-MB | 1885.404 | U/L | |  |
| MI+LY-SW-ECFCs1 | CK-MB | 1973.661 | U/L | |  |
| MI+LY-SW-ECFCs2 | CK-MB | 2295.225 | U/L | |  |
| MI+LY-SW-ECFCs3 | CK-MB | 1882.433 | U/L | |  |
| MI+LY-SW-ECFCs4 | CK-MB | 1911.429 | U/L | |  |
| MI+LY-SW-ECFCs5 | CK-MB | 2059.089 | U/L | |  |
| sham5 | CK-MB | 976.21 | U/L | |  |
| sham4 | CK-MB | 776.21 | U/L | |  |
| sham3 | CK-MB | 990.829 | U/L | |  |
| sham2 | CK-MB | 843.6 | U/L | |  |
| sham1 | CK-MB | 1053.204 | U/L | |  |

## Table S4. Serum lactate dehydrogenase (LDH) levels across experimental groups.

| 项目名称 | 项目全称 | | 原理方法 | | 方法类型 | | | 主次波长 | |
| --- | --- | --- | --- | --- | --- | --- | --- | --- | --- |
|  |  |  |  |  |  |  |  |  |  |
| LDH | 乳酸脱氢酶 | | 乳酸底物法 | | 速率法 | | | 340/405 | |
| 单位 | 测量时间(S) | | 测试用量(µl) | | | | | 测试样本量(µl) | |
|  |  |  | R1 | | | R2 | | 标准 | |
| U/L | 30-120 | | 240 | | | 60 | | 5 | |
| 样本编号 | | 项目 | | 结果 | | | 单位 | |  |
| MI+SW-ECFCs1 | | LDH | | 2029.926 | | | U/L | |  |
| MI+SW-ECFCs2 | | LDH | | 1968.943 | | | U/L | |  |
| MI+SW-ECFCs3 | | LDH | | 1837.709 | | | U/L | |  |
| MI+SW-ECFCs4 | | LDH | | 2071.516 | | | U/L | |  |
| MI+SW-ECFCs5 | | LDH | | 1736.017 | | | U/L | |  |
| MI+ECFCs1 | | LDH | | 1977.184 | | | U/L | |  |
| MI+ECFCs2 | | LDH | | 1896.13 | | | U/L | |  |
| MI+ECFCs3 | | LDH | | 1721.781 | | | U/L | |  |
| MI+ECFCs4 | | LDH | | 1967.253 | | | U/L | |  |
| MI+ECFCs5 | | LDH | | 1737.541 | | | U/L | |  |
| MI-1 | | LDH | | 1778.271 | | | U/L | |  |
| MI-2 | | LDH | | 2065.192 | | | U/L | |  |
| MI-3 | | LDH | | 1733.505 | | | U/L | |  |
| MI-4 | | LDH | | 1839.557 | | | U/L | |  |
| MI-5 | | LDH | | 1925.01 | | | U/L | |  |
| MI+LY-SW-ECFCs1 | | LDH | | 1705.418 | | | U/L | |  |
| MI+LY-SW-ECFCs2 | | LDH | | 1971.427 | | | U/L | |  |
| MI+LY-SW-ECFCs3 | | LDH | | 2122.16 | | | U/L | |  |
| MI+LY-SW-ECFCs4 | | LDH | | 2060.958 | | | U/L | |  |
| MI+LY-SW-ECFCs5 | | LDH | | 1823.338 | | | U/L | |  |
| sham5 | | LDH | | 106.21 | | | U/L | |  |
| sham4 | | LDH | | 73.897 | | | U/L | |  |
| sham3 | | LDH | | 75.594 | | | U/L | |  |
| sham2 | | LDH | | 82.776 | | | U/L | |  |
| sham1 | | LDH | | 61.467 | | | U/L | |  |

Table S5. Echocardiographic assessment of cardiac function across experimental groups.

| Parameters | Sham | MI+PBS | MI+ECFCs | MI+SW-ECFCs | MI+LY294002-SW-ECFCs |
| --- | --- | --- | --- | --- | --- |
| LVEF% | 93.07 ± 1.277 | 59.43 ± 2.888 | 72.53 ± 6.430 | 86.23 ± 4.004 | 78.92 ± 2.800 |
| LVEF(95% CIs) | [91.73, 94.41] | [56.40, 62.46] | [65.79, 79.28] | [82.03, 90.44] | [75.98, 81.85] |
| FS% | 60.50 ±2.362 | 27.65 ±1.836 | 36.77 ±5.199 | 50.40 ±5.343 | 42.23 ±2.789 |
| FS(95% CIs) | [58.02, 62.98] | [25.72, 29.58] | [31.31, 42.22] | [44.79, 56.01] | [39.31, 45.16] |
| LVIDd(mm) | 4.982±0.2385 | 7.320±0.3326 | 6.338±0.7407 | 4.925±0.8067 | 6.308±0.4875 |
| LVIDd(95% CIs) | [4.731, 5.232] | [6.971, 7.669] | [5.561, 7.116] | [4.078, 5.772] | [5.797, 6.820] |
| LVIDs(mm) | 2.070±0.3196 | 5.713±0.7549 | 4.533±0.9613 | 2.202±0.7812 | 4.112±1.006 |
| LVIDs(95% CIs) | [1.735,2.405] | [4.921, 6.506] | [3.524, 5.542] | [1.382, 3.022] | [3.056, 5.167] |
| ESV(mL) | 0.02583±0.01742 | 0.3655±0.06715 | 0.1000±0.03648 | 0.04650±0.03648 | 0.09933±0.04253 |
| ESV(95% CIs) | [0.007555, 0.04411] | [0.2950, 0.4360] | [0.06172, 0.1383] | [0.02602, 0.06698] | [0.05470, 0.1440] |
| EDV(mL) | 0.3262±0.1880 | 0.9007±0.1513 | 0.3682±0.1144 | 0.3450±0.1125 | 0.4690±0.1997 |
| EDV(95% CIs) | [0.1289, 0.5234] | [0.7419, 1.059] | [0.2481, 0.4882] | [0.2270, 0.4630] | [0.2594, 0.6786] |

| Parameters | Sham | MI+PBS | MI+ECFCs | MI+SW-ECFCs | MI+LY294002-SW-ECFCs |
| --- | --- | --- | --- | --- | --- |
| Fibrosis Area(%) | 0.2012±0.04694 | 10.74±0.7769 | 7.938±0.5369 | 2.808±0.9706 | 4.806 ± 0.6104 |
| Fibrosis Area(95% CIs) | [0.1429, 0.2595] | [9.778, 11.71] | [7.272, 8.605] | [1.603, 4.013] | [4.048, 5.564] |
| TUNEL Positive cells(%) | 2.600±1.817 | 67.40±7.092 | 46.40±7.232 | 14.60±4.615 | 32.80±3.421 |
| TUNEL Positive cells(95% CIs) | [0.3444, 4.856] | [58.59, 76.21] | [37.42, 55.38] | [8.869, 20.33] | [28.55, 37.05] |
| TUNEL Positive cells (fold of control) | 1.000±0.5900 | 18.41±0.8787 | 14.02±2.245 | 4.647±1.095 | 8.991±1.972 |
| TUNEL Positive cells (fold of control) (95% CIs) | [0.2674, 1.733] | [17.32, 19.50] | [11.23, 16.81] | [3.288, 6.006] | [6.543, 11.44] |
| α-SMA+ cells (Area%） | 1.150±0.06014 | 0.7929±0.1210 | 1.342±0.06351 | 1.669±0.1191 | 1.407±0.07408 |
| α-SMA+ cells (95% CIs) | [1.076, 1.225] | 0.6426, 0.9432] | [1.263, 1.421] | [1.521, 1.817] | [1.315, 1.499] |
| CD31+ cells (Area%） | 1.119±0.1161 | 0.7476±0.1164 | 1.219±0.1224 | 1.896±0.2160 | 1.507±0.1306 |
| CD31+ cells(95% CIs) | [0.9751, 1.263] | [0.6031, 0.8921] | [1.067, 1.371] | [1.628, 2.164] | [1.345,1.669] |
| Positivity rate of p-eNOS(Area%) | 2.172±0.2158 | 0.2820±0.07855 | 0.5680±0.1663 | 0.9760±0.1176 | 0.6380±0.1033 |
| Positivity rate of p-eNOS(95% CIs) | 1.904,2.440] | [0.1845, 0.3795] | [0.3615, 0.7745] | [0.8300, 1.122] | [0.5097, 0.7663] |
| VEGF-A^+^ cells (fold of control) | 1.000±0.2758 | 2.401±0.3760 | 5.470±0.7836 | 10.08±1.191 | 3.837±0.4541 |
| VEGF-A^+^ cells (fold of control)(95% CIs) | [0.6575, 1.342] | [1.934, 2.867] | [4.497, 6.443] | [8.601, 11.56] | [3.273, 4.401] |

Table S6. Histopathological analysis of myocardial tissue across experimental groups.

Table S7. Western blot analysis of PI3K/AKT pathway-related proteins in myocardial tissue across experimental groups.

| Parameters | Sham | MI+PBS | MI+ECFCs | MI+SW-ECFCs |
| --- | --- | --- | --- | --- |
| p-AKT/AKT | 1.251±0.2392 | 0.3855±0.1287 | 0.8324±0.2600 | 1.221±0.1882 |
| p-AKT/AKT (95% CIs) | [0.9995,1.502] | [0.2505,0.5206] | [0.5596,1.105] | [1.023,1.418] |
| p-eNOS/eNOS | 1.169 ±0.2492 | 0.3911 ±0.1232 | 0.7939 ±0.1405 | 1.150 ±0.2364 |
| p-eNOS/eNOS (95% CIs) | [0.9080, 1.431] | [0.2618, 0.5204] | [0.6465, 0.9413] | [0.9021, 1.398] |
| Cleaved caspase-3/GAPDH | 0.4862±0.1898 | 1.325±0.1124 | 0.8122±0.1574 | 0.5544±0.07602 |
| Cleaved caspase-3/GAPDH (95% CIs) | [0.2871,0.6854] | [1.207, 1.443] | [0.6471, 0.9774] | [0.4747, 0.6342] |
| BCL-2/GAPDH | 1.462±0.2190 | 0.3999±0.1384 | 0.8067±0.2212 | 1.350±0.3619 |
| BCL-2/GAPDH (95% CIs) | [1.232, 1.692] | [0.2547, 0.5452] | [0.5745, 1.039] | [0.9704, 1.730] |
| NO content in cardiac tissue | 0.4961±0.04001 | 0.1312±0.02953 | 0.2080±0.03252 | 0.4062±0.05174 |
| NO content in cardiac tissue (95% CIs) | [0.4541, 0.5381] | [0.1002, 0.1622] | [0.1739, 0.2421] | [0.3519, 0.4605] |
| NO content in plasma | 0.1901±0.01576 | 0.02422±0.008746 | 0.04376±0.007187 | 0.1126±0.007330 |
| NO content in plasma (95% CIs) | [0.1736, 0.2067] | [0.01504, 0.03340] | [0.03621, 0.05130] | [0.1049, 0.1203] |

Tables S8 Western blotting analysis of heart tissue from different groups.

| Parameters | MI+PBS | MI+SW-ECFCs | MI+LY294002-SW-ECFCs |
| --- | --- | --- | --- |
| p-AKT/AKT | 0.6032 ± 0.06030 | 0.9925±0.03405 | 0.7856 ±0.1657 |
| p-AKT/AKT (95% CIs) | [0.5399, 0.6665] | [0.9568, 1.028] | [0.6117, 0.9595] |
| p-eNOS/eNOS | 0.4911 ± 0.2460 | 1.206 ±0.2171 | 0.8814 ± 0.1372 |
| p-eNOS/eNOS (95% CIs) | [0.2328, 0.7493] | [0.9782, 1.434] | [0.7374, 1.025] |
| Cleaved caspase-3/GAPDH | 1.030±0.1807 | 0.3525±0.09113 | 0.7818±0.1959 |
| Cleaved caspase-3/GAPDH (95% CIs) | [0.8401, 1.219] | [0.2569, 0.4481] | [0.5762, 0.9874] |
| BCL-2/GAPDH | 0.6022±0.1897 | 1.360±0.2704 | 0.9964±0.1324 |
| BCL-2/GAPDH (95% CIs) | [0.4032, 0.8013] | [1.077, 1.644] | [0.8575, 1.135] |
| NO content in cardiac tissue | 0.1312±0.02953 | 0.4062±0.05174 | 0.2746±0.02138 |
| NO content in cardiac tissue (95% CIs) | [0.1002, 0.1622] | [0.3519, 0.4605] | [0.2522, 0.2971] |
| NO content in plasma | 0.02422±0.008746 | 0.1126±0.007330 | 0.05936±0.003988 |
| NO content in plasma (95% CIs) | [0.01504, 0.03340] | [0.1049, 0.1203] | [0.05517, 0.06354] |
